# Bioinspired Adaptive Leg‐Claw Enables Robust Perching and Grasping for UAVs

**DOI:** 10.1002/advs.202523518

**Published:** 2026-03-13

**Authors:** Tianyu Cheng, Shaokun Wang, Jiehan Zou, Guiwen Shi, Zhongxue Gan, I‐Ming Chen, Guo‐Niu Zhu

**Affiliations:** ^1^ College of Intelligent Robotics and Advanced Manufacturing Fudan University Shanghai China; ^2^ School of Mechanical and Aerospace Engineering Nanyang Technological University Singapore Singapore

**Keywords:** adaptive leg‐claw system, aerial manipulation, bioinspired robotics, tensile structure, UAV

## Abstract

Conventional unmanned aerial vehicles (UAVs) are limited in their ability to perch and grasp, constraining aerial manipulation and prolonged operation in complex environments. Raptors, however, achieve versatile and robust functions through specialized hindlimb morphologies. Inspired by the owl's limb structure and the bat's roosting behavior, we propose a bioinspired adaptive leg‐claw mechanism that enables UAVs to perform robust and versatile perching and grasping. Our design integrates a four‐link tibia, tension‐driven deformable feet, and symmetrical toes to ensure stable attachment to surfaces of varying geometry. An active grasping strategy and control framework are developed to enable autonomous switching between standing and hanging perching modes, and adaptive toe adjustment for grasping diverse objects. The design allows UAVs to seamlessly transition between perching modes, land on branches of different diameters, and grasp irregular objects with stability. The bioinspired mechanism demonstrates significant potential for enabling UAVs to perch and grasp reliably in unstructured environments.

## Graphical abstract

Inspired by owl limb morphology and bat roosting behavior, this study presents a bioinspired adaptive leg‐claw mechanism that enables UAVs to perform robust and versatile perching and grasping. The design integrates a four‐link tibial structure, tension‐driven deformable feet, and active control, enabling stable perching on various branches and adaptive grasping of irregular objects.

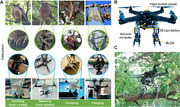

## 1 Introduction

Raptors exhibit remarkable perching and grasping capabilities enabled by their distinctive hindlimb morphology. They can maintain stable perching on diverse and irregular surfaces and capture prey of varying sizes with precision [1]. In natural habitats, raptors perch on branches, rocks, and the ground, where geometric characteristics vary considerably. In contrast, unmanned aerial vehicles (UAVs) typically rely on landing gear for takeoff and landing, which restricts the range of perching sites and prevent active grasping [2, 3, 4, 5, 6, 7]. Although recent studies have sought to enhance UAV perching performance, most remain constrained by the geometry of the target surfaces and exhibit limited adaptability [8, 9, 10, 11, 12] (Table S1). Attempts to integrate landing gear design with raptor‐inspired morphologies are still in an exploratory stage [13].

The powerful perching and grasping abilities of raptors are governed by two key mechanisms in their legs: the digital flexor mechanism (DFM) and the tendon locking mechanism (TLM) [14, 15]. The DFM drives claw closure during leg flexion, while the TLM locks the toes in place to provide additional grip strength. Raptors also possess highly flexible claws capable of small rotational adjustments when perched. This flexibility allows the toe morphology to adapt to surface geometry: a parallel configuration is suited for cylindrical objects, whereas a nonparallel configuration is effective for flat or irregular surfaces [1, 16, 17]. Elements of the DFM and TLM have been incorporated into certain bioinspired designs [18, 19], and rotatable toes have been explored in robotic grippers [20, 21, 22]. However, the application of these mechanisms to UAV perching remains at an early stage [23].

Although raptors exhibit excellent perching ability, they can perch only on relatively large surfaces. Perching becomes unstable or fails when the available surface area is insufficient to generate adequate frictional support. This limitation is also present in most biomimetic perching mechanisms. Consequently, researchers have explored integrating raptor‐inspired strategies with other natural perching approaches, which can be categorized into three types: grasping, embedded, and attached [24, 25, 26, 27, 28]. Most bioinspired perching mechanisms utilize grasping due to its reliability for larger and heavier multirotor UAVs. Current grasping designs fall into two categories. The first is a raptor‐inspired standing perching mechanism, which offers low energy consumption and rapid escape initiation. However, this design imposes a strict minimum branch diameter requirement to ensure stable perching [18, 29, 30, 31, 32, 33]. The second is a bat‐like hanging perching mechanism, which minimizes visual obstruction and uses gravity for passive stabilization. Conversely, this approach requires branches below a specific diameter [34, 35, 36, 37, 38]. Both mechanisms rely on the TLM for stability and complement each other across roosting target sizes. Recent work has coupled these mechanisms to enable UAVs to perform both standing and hanging perching [39]. However, UAVs must be fully hanging like a bat to achieve hanging perching, which can complicate control and make it difficult for heavier UAVs to take off in a flip maneuver.

In addition to their perching ability, raptors are also notable for their rapid grasping, which enables them to capture objects of varied shapes within a short time [40, 41]. Arboreal raptors typically have short digits, yet their tendon system allows them to firmly seize and subdue prey with their talons [42, 43]. Many studies have investigated UAV grasping capabilities, often without addressing their perching functionality [44, 45, 46, 47, 48]. Although some works have integrated grasping into perching mechanisms, most designs rely on passive triggering, which requires specific impact forces to activate the grasp. Furthermore, these systems lack active control, which significantly limits grasping reliability in real‐world environments and constrains the multifunctional potential of UAVs [19, 34].

Inspired by raptors, we propose a novel UAV perching mechanism that enables both standing and hanging perches without requiring a flip maneuver. Based on the inspiration from the morphology of owl hindlimbs and the perching behavior of bats, we present a bioinspired leg‐claw mechanism (BLCM) for UAV perching and grasping. This mechanism represents an innovative architectural integration of established engineering concepts rather than the introduction of new physical principles or theoretical models. Our design consists of a tibia with a four‐bar linkage [49], deformable feet based on tension‐driven structures [50, 51, 52, 53], and symmetrical toe rows. The tibia's compression converts the UAV's weight into grip force during perching, thereby reducing energy expenditure. The deformable feet, with their tensioning structure, allow the BLCM to passively adapt to various surfaces, such as cylinders, flat surfaces, and pits. The toe angle can be actively adjusted or passively accommodated. Each toe is equipped with high‐friction rubber pads to ensure reliable perching and grasping. The BLCM incorporates active claw closure control for precise perching and object grasping. Additionally, independent lateral swing joints enable leg rotation without full drone inversion, improving safety and operational simplicity during hanging perching. The BLCM can also adapt its toe configuration to target geometry to ensure optimal perching and grasping. It enables stable perching on objects of varying diameters and shapes without altering the UAV's attitude. The system automatically switches between standing and hanging perching modes. We evaluate our design through multiple perching and grasping experiments. The results demonstrate that our proposed bioinspired design allows UAVs to transition between perching modes, land on branches of different diameters, and grasp diverse objects with stability. This architectural integration renders the BLCM highly promising for UAV perching and grasping in unstructured environments. It enables stable perching on branches with a wide range of diameters, across diverse surface geometries, and in both standing and hanging modes without requiring drone inversion, thereby demonstrating strong potential for reliable UAV perching and grasping in complex real‐world environments.

## 2 Results

### 2.1 Bioinspired Design for an Adaptive UAV Perching Mechanism

**FIGURE 1:**
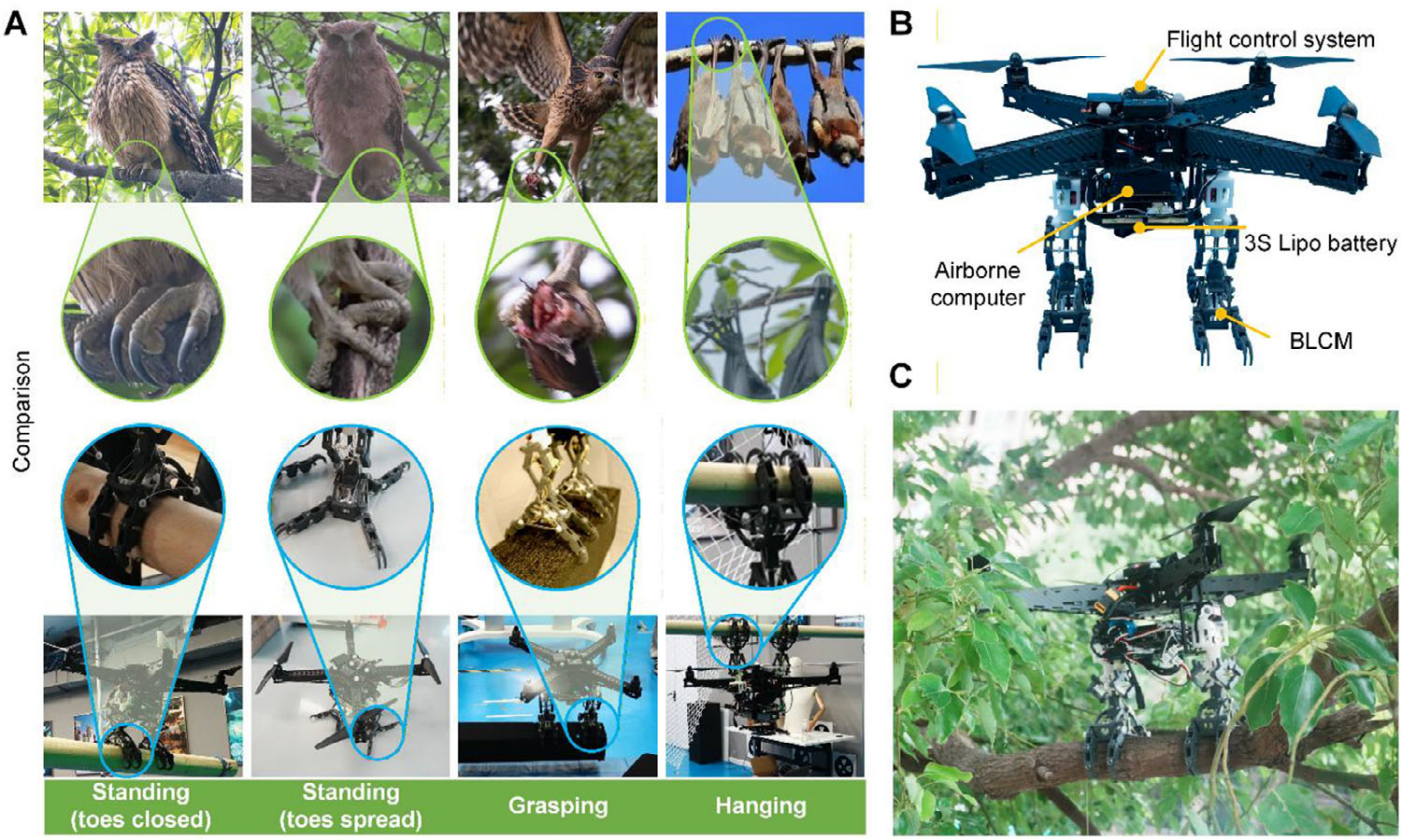
The bioinspired leg‐claw mechanism (BLCM) for quadrotor UAVs. (A) Comparative morphology of owls (*Ketupa flavipes*) and bats. Owls perch and grasp using their zygodactyl hindlimbs, whereas bats achieve inverted hanging perching through their talons. UAVs equipped with BLCM inherit both standing and hanging perching capabilities. (B) Integration of BLCM onto a quadrotor platform, illustrating the scalability of the mechanism with respect to UAV size. (C) Demonstration of BLCM in field application: the UAV successfully perches on a tree branch (∼75 mm diameter, 5° tilt).

The BLCM prototype is inspired by the robust legs and claws of owls, which enable perching on diverse surfaces and grasping prey during flight. Owls possess zygodactyl toes, with two oriented forward and two backward, whose angles can be actively or passively adjusted to achieve a compliant grip on various objects [52, 54]. The design further incorporates the hanging perching strategy of bats [55, 56], in which joint rotation allows the legs to flip into a suspended posture. The perching patterns of BLCM are compared with those of owls and bats (Figure 1A), and a UAV equipped with the BLCM is illustrated in Figure 1B,C.

**FIGURE 2:**
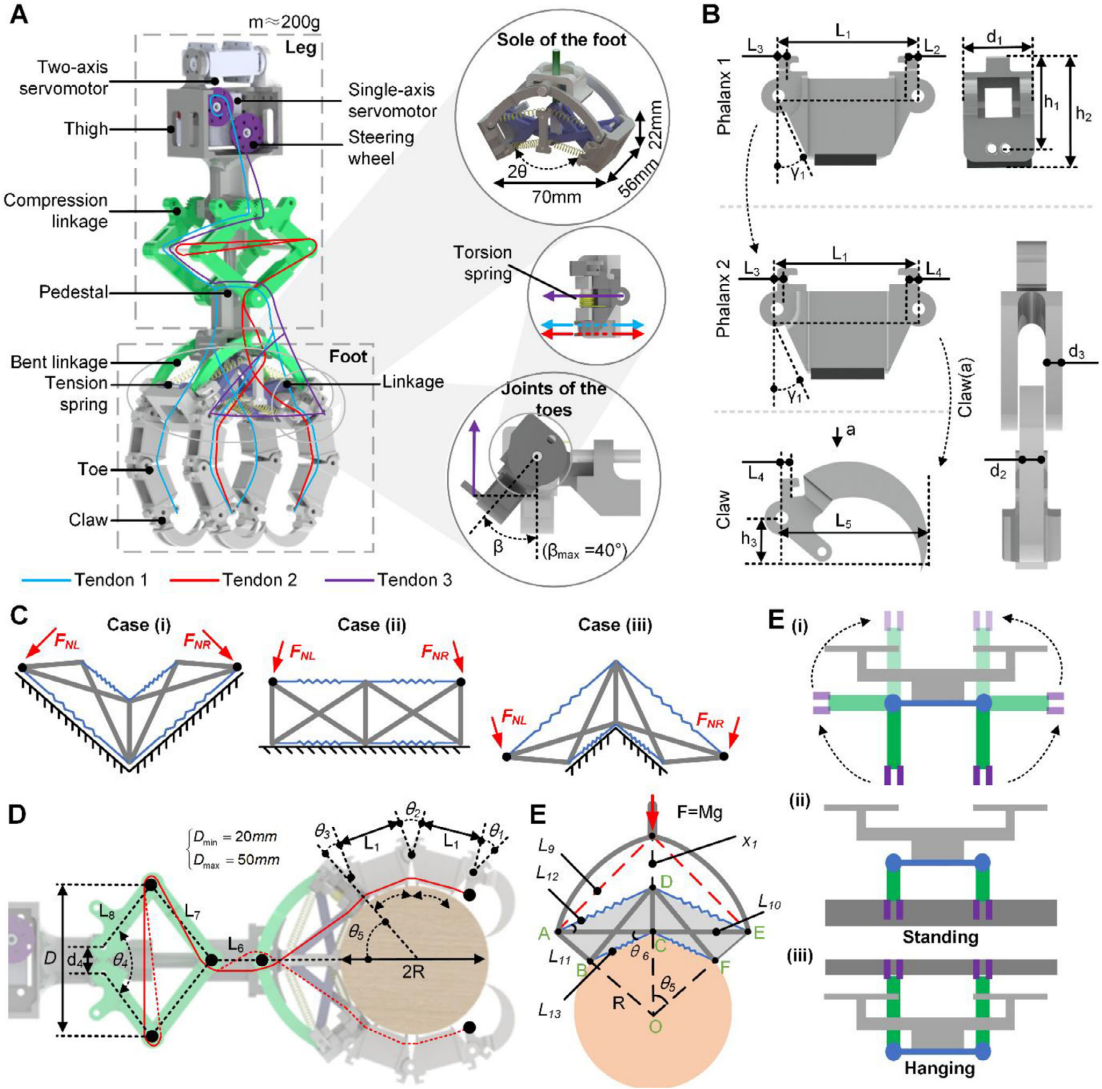
Bioinspired design and working principle of the owl‐inspired perching mechanism. (A) Overall structure of BLCM. The blue solid line denotes the tendon that actively controls claw closure. The red solid line denotes the passive tendon. The purple solid line denotes the tendon that actively controls toe motion. (B) Design parameters of the toe and claw. (C) Principles of tensile structures are shown, with three cases demonstrating their adaptability to different shapes. (D) The claws passively adapt to the branches. (E) Switch between hanging and standing postures by rotating the entire BLCM.

BLCM consists of three components: (i) a tensile foot structure that passively adapts to surfaces with varying curvatures, (ii) a compressible outrigger based on a four‐bar linkage that mimics the digital flexor mechanism (DFM) of birds [57], and (iii) symmetrically arranged underactuated toes that squeeze and grasp the target. All three components adopt an underactuated design, which achieves shape adaptation to various objects with a minimal number of driving degrees of freedom [19]. BLCM's toes are simplified into a fully symmetrical configuration (Figure 2A), with each toe comprising a rotation joint, two equal‐length phalanges, and a claw. The joints permit rotation angles between 0° and 40°. For cylindrical objects, the inter‐toe angle approaches 0°, whereas flatter objects require larger angles, thereby improving grasping stability. All toes are equal in length (Figure 2B), scaled according to both UAV and owl toe dimensions, and use identical rubber bands to provide return forces. To enhance grasping efficiency, the joints closer to the foot bend earlier, achieved by adjusting the preload of the rubber bands (*L_2_* < *L_3_* < *L_4_*). The toes are mounted on the foot, which is designed to match UAV dimensions and composed of two serially connected X‐structures, each consisting of four linkages and four tension springs (Figure S1). The X‐tensile structure has three discrete states that adapt to surfaces of different shapes (Figure 2C). It exhibits anisotropic stiffness: bending is allowed in one direction while higher stiffness is maintained in others. This property reduces the likelihood of lateral failure and ensures both structural safety and functional reliability.

The paw is connected to the leg mounting base through two sets of bent linkages. One end of each linkage is concentrically fixed, while the other end is attached to two shafts above the paw. When a compressive load is applied from above, the linkages transfer the force to both sides of the paw, inducing a bending motion (Figure 2D). This mechanism enables passive adaptation of the paw to the curvature of the contacted surface. The length of the bent linkages directly influences the closing force: shorter linkages generate larger pinch angles and greater pressure. This configuration lowers the center of gravity and increases gripping force, albeit at the cost of reducing the flexion range of the foot. Consequently, the shortest feasible bent linkage length is adopted, as it satisfies the required flexion angle. Detailed calculations for determining the linkage length are provided in Materials and Methods.

The legs of BLCM consist of a shin and a femur. The femur is used to attach the bottom end of the vehicle and to mount the servos. The structural element of the shin is a four‐bar linkage that enables passive stretching of the tendon. Beneath the mechanism is the base that connects to the leg. During branch grasping, the two upper connecting rods are gear‐meshed to maintain equal angles relative to the central axis. A movable shaft at the center of the leg links the femur to the base, ensuring that the leg axis remains perpendicular to the ground. Compression of the four‐bar linkage increases the horizontal distance between the two joint edges, elongating the leg tendon and thereby driving the claw to close. For the drone to achieve passive perching, the maximum tendon elongation must exceed the shortening produced during branch grasping. The relationship is expressed as follows:

(1) $$L_{leg}^{1}-L_{leg}^{0}>L_{foot}^{0}-L_{foot}^{1}$$

where $L_{\mathrm{leg}}^{1}$ is the maximum length of the leg tendon, $L_{\mathrm{leg}}^{0}$ is the initial tendon length, $L_{\mathrm{foot}}^{1}$ is the tendon length within the toes when the foot is unflexed, and $L_{\mathrm{foot}}^{0}$ is the tendon length during branch grasping. A larger initial value of *θ_4_*, the angle of the upper linkage, facilitates leg bending. However, increasing *θ_4_* also enlarges the leg width, thereby raising the risk of collision with the wing during folding. Thus, the following condition must be satisfied.

(2) $$2L_{9}\sin\frac{\theta_{4}}{2}+d_{4}\leq D_{\min}$$

where *D_min_* is the minimum distance between two adjacent wings of the UAV. The change in tendon length is related to leg dimensions.

(3) $$\begin{array}{c} \left\{\begin{array}{c} 2L_{9}\left(\sin\frac{\theta_{4}}{2}-\sin\frac{\theta_{4\min}}{2}\right)=L_{foot}^{0}-L_{foot}^{1}\\ L_{foot}^{0}-L_{foot}^{1}=\frac{3L_{1}\left(R+h_{2}-h_{1}\right)}{2\left(R+h_{2}\right)}+\frac{\sqrt{{R_{2}}^{2}+{\left(L_{10}/2+L_{5}\right)}^{2}}-R}{L_{6}} \end{array}\right.\;\;\; \end{array}$$

From Equation (3), *θ_4_* can be determined, allowing the change in leg height to be calculated for branches of any diameter. In addition, when the claw is fully closed, the tendon shortening is approximately 38 mm, corresponding to a maximum servo rotation angle of about 385°.

The prototype is mounted on a UAV with a 450 mm wheelbase and a total takeoff weight of approximately 1.2 kg (recommended range, 1–1.5 kg), including the battery but excluding the landing gear (Figure S2). The landing gear weighs about 430 g, with each leg weighing 200 g and incorporating three servos (63 g total). The control board and wiring add an additional 30 g. Overall, the landing gear accounts for ∼24.7 % of the total vehicle mass, consistent with results reported [58]. Various perching modes of the UAV equipped with the BLCM are demonstrated in Figure 2E. The dimensions of the major components are summarized in Table S2, and details of manufacturing, assembly, and control are provided in Methods.

### 2.2 Assessment of Passive Standing Perching Performance

BLCM enables hawk‐like standing perching, a style suitable for diverse surfaces and relatively thick branches. This mode relies solely on UAV weight for passive engagement: as the vehicle lands top‐down on a branch, gravity induces leg bending that drives the claw to close. High‐friction material at the leg bases increases contact force, thereby securing the UAV to the branch. However, leg tilting caused by external disturbances, positioning errors, or other factors may compromise stability, leading to perching failure and potential falls. We further examine the relationship between balance failure, applied load, and tilt angle to address this risk.

When the UAV perches on thick branches, gravity generates large squeezing forces *F_N1_* and *F_N3_* (Figure S3). A smaller squeezing force *F_N2_* arises at the paw center due to the spring return force. Gravity also induces a substantial tendon tensile force *F_T_*, which in turn produces a large claw‐squeezing force against the branch, *F_N5_*. This force generates a reverse moment at the claw‐to‐toe joints, causing outward bulging of the toe joints. As a result, the direct squeezing force of the toes on the branch is minimal and can be neglected. This observation is consistent with findings reported for avian species [59].

**FIGURE 3:**
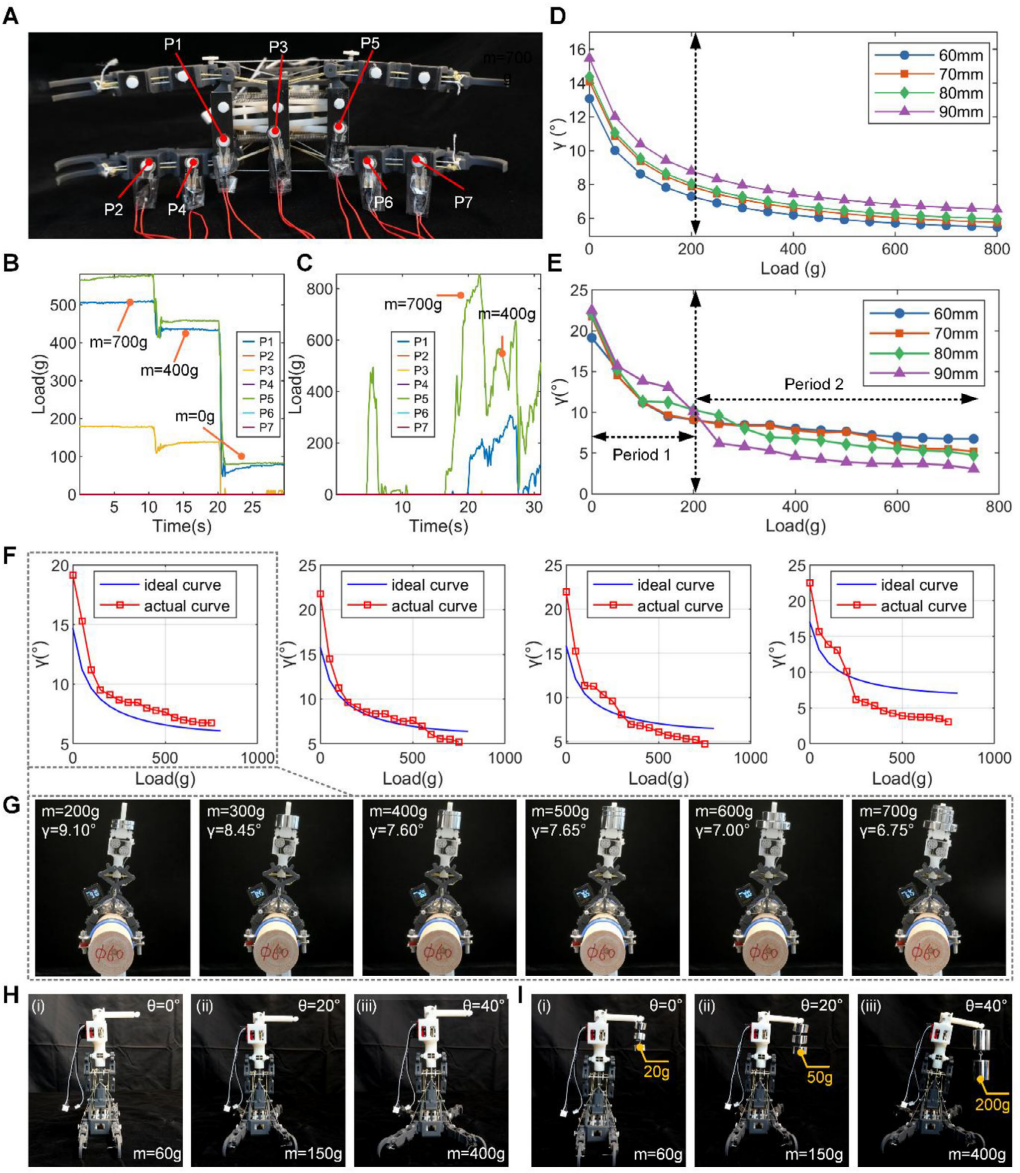
Schematic and experimental results of BLCM standing perching. (A) Mounting positions of seven strain gauges on the underside of the foot, all installed on the same side. (B) Force curves measured at various locations on the sole and toe bottoms under different loads (700, 400, and 0 g) when the mechanism perches vertically on a branch. (C) Force curves measured at various locations on the sole and toe bottoms under different loads (700, 400, and 0 g) when the mechanism perches on an inclined branch. (D) Theoretical relationship between load and maximum tilt angle for branches of varying diameters (60, 70, 80, 90 mm). (E) Experimental relationship curves between load and maximum tilt angle across different branch diameters. (F) Comparison of theoretical and experimental load‐tilt angle relationships for branches of varying diameters. (G) Critical tilt angle tests on a 60 mm‐diameter branch, with increasing loads from left to right. (H) BLCM perched on the ground with toe angles of 0°, 20°, and 40°, respectively. (I) Maximum supported load for three different toe‐clamp angle configurations.

In order to validate the distribution pattern of pressure, seven thin‐film pressure sensors are installed on one side of the toes and at the foot base to measure pressure distribution (Figure 3A). All sensors are calibrated and connected to a signal conversion module prior to use (see Methods for details). To ensure consistent loading, small discs with an area equal to the sensing surface are mounted on the sensors, with symmetrical discs placed on the opposite side. Sensor signals are aggregated via two four‐channel converter modules and transmitted to a PC for visualization and analysis.

At tilt angles of 0° and 10°, a 700 g load is applied, after which 300 g is removed, followed by complete unloading. The corresponding pressure variations are recorded (Figure 3B,C; Figure S4). At 0°, pressures are concentrated at the ball of the foot, with *F_N1_* and *F_N3_* dominating and exhibiting nearly equal magnitudes at positions q1 and q5. Pressure at q3 is smaller, while the remaining positions are negligible. At 10°, the pressure distribution remains foot‐centered, but the central load decreases further, producing an imbalance between the two sides of the foot. The experimental results are consistent with theoretical predictions. Therefore, toe pressure and friction effects are neglected in subsequent analysis. Under these conditions, the equilibrium relationship between moment *M* and tilt angle *γ* is derived (see Methods).

We measure *M*‐*γ* curves for branches of different diameters. This is done to verify the relationship between load and maximum tilt angle. Since standing perching requires branch diameters of at least 50 mm, pine branches with diameters of 60, 70, 80, and 90 mm are selected for testing. Given the UAV's maximum mass of 1.5 kg, the maximum unilateral hindlimb load is 750 g. Therefore, test loads range from 0 to 750 g and are determined by interpolation.

During testing, the leg is vertically aligned using a level, and an inclinometer is mounted proximally to the foot to record tilt via angular displacement. Under controlled loading, a gradual medial‐lateral force is applied to the proximal femur until collapse occurred, at which point the critical tilt angle is recorded. Each condition is repeated three times, and mean values are reported (Table S3). Theoretical predictions are then compared with experimental results (Figure 3D,E).

Theoretical analysis predicts that, for a given load, the maximum perching angle should increase with branch diameter. However, this trend is not consistently observed in practice. At smaller loads, experimental results agree more closely with theory, whereas at loads exceeding 200 g, the maximum perching angle is greater on a 60 mm branch (Figure 3E). To clarify this discrepancy, we compare theoretical and experimental curves (Figure 3F,G). At low loads, the two curves exhibit similar trends with a slight offset, primarily due to the omission of frictional effects in the theoretical model (Figure 3F). As branch diameter increased, a critical load threshold emerged beyond which the maximum perching angle drops markedly below theoretical predictions. Moreover, the critical point shifts toward lower loads with increasing branch diameter. According to Equation (3), this behavior arises because larger diameters reduce tendon contraction distance while significantly increasing leg length. The elongated legs elevate the center of gravity, which reduces stability. They also induce greater bending along the leg axis, with longer legs showing more pronounced deformation. (Figure 3G). These findings indicate that thinner branches provide more favorable conditions for stable standing perching. In our tests, the UAV (1.2 kg without landing gear) tolerates ±5.5° of axial tilt on branches 60–80 mm in diameter.

The BLCM may tilt laterally under eccentric loading while perching on the ground [60]. We test the unilateral hindlimb under eccentric forces (Figure 3H,I). A 60 mm beam is mounted on the top of the leg, from which a mass could be suspended. Toe pinch angles are fixed at 0°, 20°, and 40°, and hook weights are incrementally added to the beam end until imbalance occurs. The critical mass is recorded at this point. The results demonstrate that at a toe angle of 0°, the maximum load is 60 g, whereas at 40° the maximum load increases to 400 g, representing an improvement of ∼567 %. These findings indicate that larger toe pinch angles substantially enhance perching stability. Moreover, owing to the flexibility of the fabrication material, the leg exhibits minor bending and tilting under large eccentric forces but does not incur structural damage.

While the present BLCM demonstrates reliable passive standing perching, its stability is inherently limited by the tilt angle of the target surface. To extend the feasible perching threshold, active reinforcement strategies can be employed. For instance, the tendon‐driving servos can provide supplementary active squeezing force to increase the friction limit when a large tilt is detected. These active interventions, combined with the existing high‐friction rubberized contact points, would allow the BLCM to operate in even more extreme unstructured environments.

### 2.3 Hanging Perching Performance and Power Optimization

The BLCM also demonstrates hanging perching, analogous to the behavior of bats. This strategy is particularly effective for grasping slender structures such as ropes and small branches. Hanging perching requires a 180° rotation of the legs, orienting the soles upward, after which the UAV approaches the target from below. Upon contact, the servo actuates the tendons to close the foot and achieve suspension.

**FIGURE 4:**
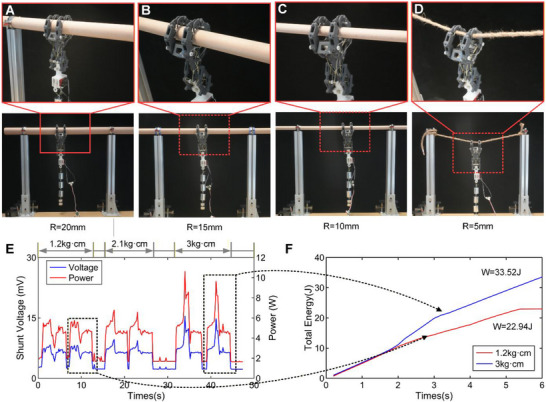
Hanging perching experiments and power optimization of BLCM. (A–D) Demonstration of hanging perching on objects with four different diameters. (E) Power and voltage curves of the BLCM during multiple grasping operations under different output torque conditions. (F) Total energy consumption of the BLCM before (3 kg·cm) and after (1.2 kg·cm) optimization. The average total energy required for a complete grasping task decreased from 33.52 J (6.0 s) to 22.94 J (5.6 s), corresponding to a ∼31.7 % reduction in total energy loss.

Compared with standing perching, hanging perching eliminates concerns of instability caused by shifts in the center of gravity or insufficient friction. The primary challenge, however, lies in preventing failure due to inadequate grip. Under equivalent output conditions, the following requirements must be satisfied:

(4) $$\left\{\begin{array}{c} P=F_{out}\cdot v\\ T_{out}=F_{out}\cdot L_{0} \end{array}\right.$$

where *L_0_* denotes the force arm, *F_out_* is the output force, and *T_out_* is the output torque. The servo output torque should be larger if the grip force is to be improved. However, an increase in torque results in a substantial rise in both power output and energy consumption. Thus, minimizing output torque while maintaining perching safety is essential. The critical torque at the maximum load is determined experimentally using four objects of different diameters under a 750 g load. In these tests, the servo output torque was initially set to 3.0 kg·cm and then decreased stepwise in increments of 0.15 kg·cm until 1.05 kg·cm was reached (Figure 4A–D). The results indicate that stable and energy‐efficient perching can be achieved with a servo torque of 1.2 kg·cm across all tested diameters.

The power consumption of the BLCM during grasping is measured (see Methods for calculation details). Two grasping tests are performed at output torques of 1.2, 2.1, and 3.0 kg·cm, with 3.0 kg·cm representing the baseline configuration prior to optimization. In this study, “grasp” is defined as the complete cycle from the initiation of tendon actuation to full release. The corresponding power and voltage curves are shown in Figure 4E, indicating that each grasp‐release cycle requires approximately 5.6 s. This duration remains consistent across torque settings.

The total energy consumption before (3.0 kg·cm) and after (1.2 kg·cm) optimization is summarized in Fig. 4F. The results demonstrate a reduction of 10.58 J, corresponding to a 31.7 % decrease in energy dissipation. Notably, reducing torque not only decreases instantaneous power demand but also substantially lowers cumulative energy consumption over repeated trials. Such efficiency improvements are particularly relevant for UAV operations, where flight time and endurance are constrained by limited onboard energy. By reducing energy loss during perching tasks, BLCM extends operational range and provides greater flexibility for missions requiring prolonged aerial deployment or frequent perching maneuvers.

### 2.4 Powerful Active Grasping Performance

A common strategy for active grasping is to define a grasping range commensurate with the target object size. However, estimating the size of the object inevitably increases system complexity. Moreover, an excessively wide grasping range may compromise grasping stability, whereas an insufficient range can lead to excessive force when the claw contacts the object, potentially damaging both the mechanism and tendons. To address these challenges, we adopt a force‐position hybrid control strategy. In this mode, a fixed tendon contraction length is first established to ensure that the claw can fully close without necessarily enclosing the target. In parallel, an upper force threshold (*F_max_*) is set to protect the mechanism. In addition, when the target is noncylindrical, grasping stability can be enhanced by adjusting the toe pinch angle [45].

**FIGURE 5:**
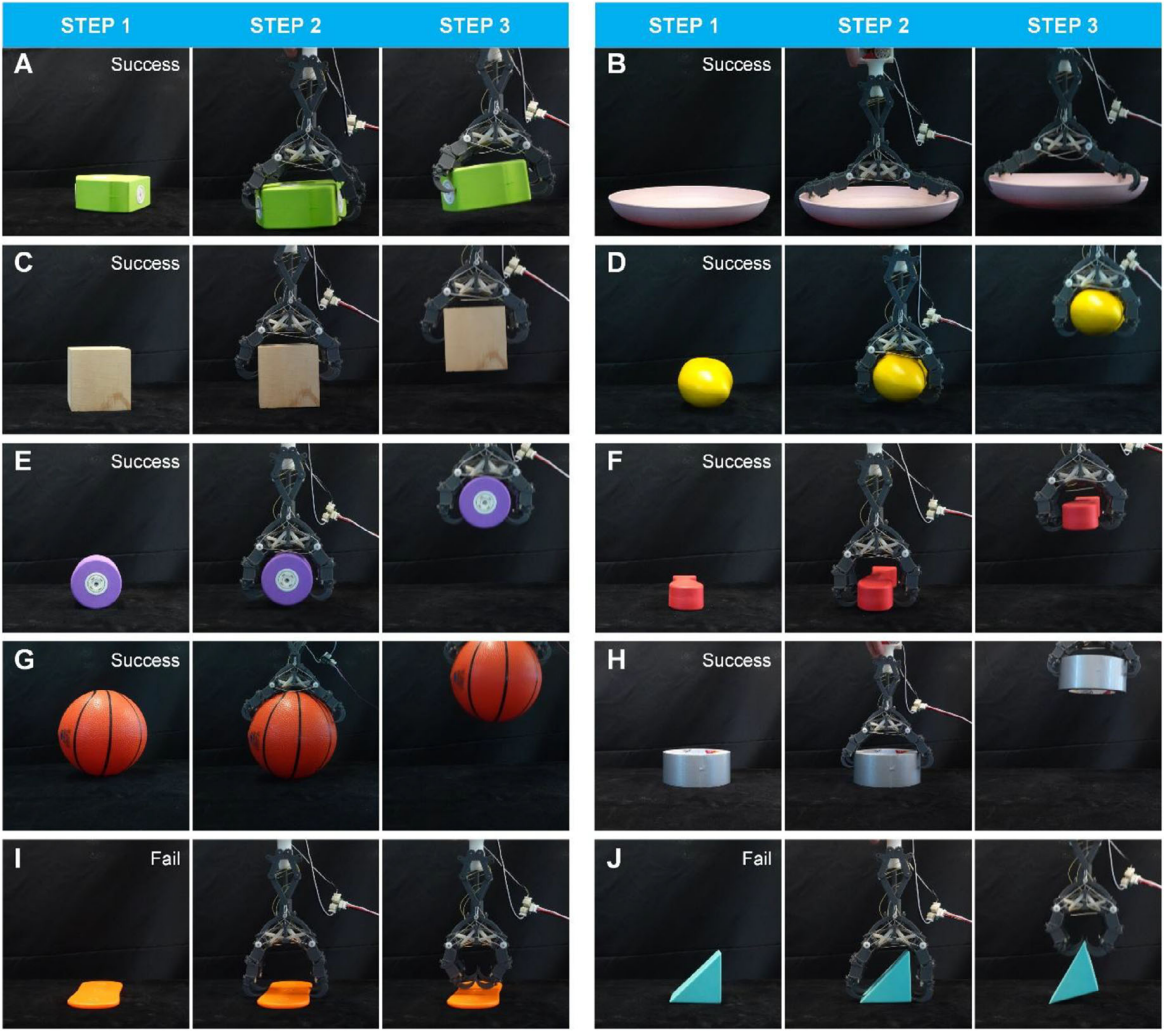
Grasping tests of the BLCM on various objects. Grasping successes for (A) Hexagonal prism, (B) plate, (C) wooden cube, (D) toy mango, (E) cylinder, (F) fish‐shaped toy, (G) leather ball, and (H) duct tape. Grasping fails for (I) sheet and (J) triangular prism.

The grasping performance of the BLCM is evaluated using 30 objects with diverse shapes, sizes, weights, and surface textures, including tools, fruit models, and small toys (Table S4). Details of the experimental setup are provided in Methods. BLCM demonstrates a high degree of reliability, successfully grasping 28 out of 30 test objects (Movie S1), corresponding to a success rate of ∼93 % (Figure 5A–H). Compared with results reported in existing studies [21, 29, 30], BLCM can grasp a broader range of objects and achieves a higher grasping success rate. This advantage is primarily attributed to its tension‐driven deformable feet and high‐friction toe pads, which enhance adaptability to diverse targets and improve overall grasping reliability.

Two objects fail to meet the grasping criteria (Movie S2), highlighting geometry as a key factor influencing grasping performance. These failure cases share a common structural characteristic: a lack of protruding edges or elevated corner features relative to the supporting surface. This deficiency prevents the claws from effectively penetrating the gap between the object and the desktop to establish stable contact points, thereby inhibiting the generation of sufficient grasping force. Overall, the BLCM demonstrates high reliability for objects with symmetrical profiles, prominent edges, or soft deformable surfaces, as these features facilitate stable enclosure and balanced force distribution. In contrast, featureless objects, such as the thin sheet or prism (Figure 5I,J), present significant challenges for secure attachment and may necessitate auxiliary grasping techniques to achieve operational success.

Beyond the static success rate, the results also underscore the versatility of BLCM design. Its ability to handle diverse object geometries without preprogrammed object recognition reduces system complexity and highlights the potential of the BLCM for general‐purpose aerial manipulation. Such versatility is particularly advantageous for UAV tasks involving unstructured environments, where variability in object geometry and surface conditions presents significant challenges.

### 2.5 Integration With UAVs for Aerial Perching and Grasping

**FIGURE 6:**
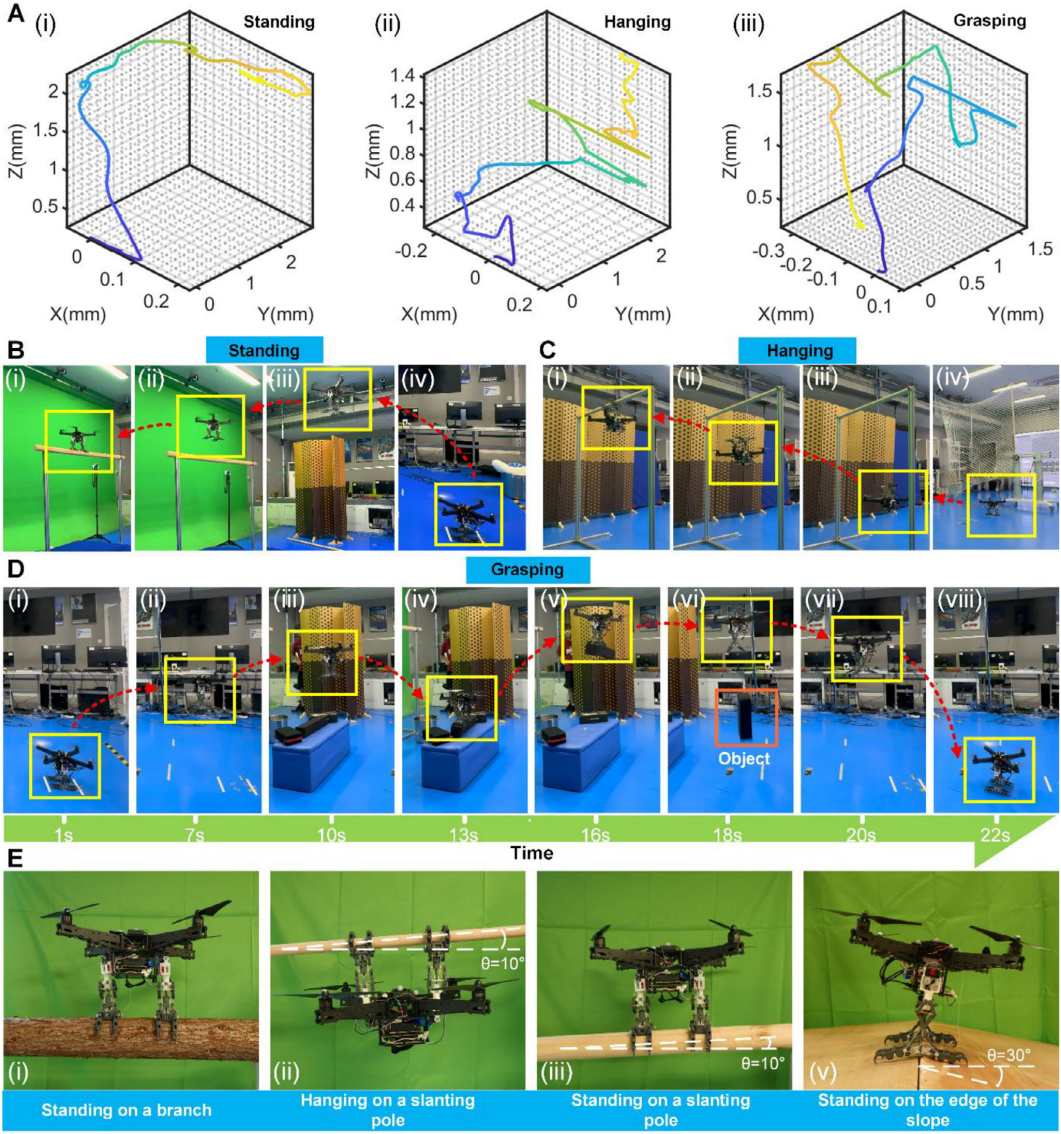
UAV perching and grasping experiments. (A) 3D trajectory curves of UAV position during standing perching, hanging perching, and grasping experiments, with all position data obtained from UAV flight control log files. (B) Snapshots of the standing perching experiment, showing the process from takeoff to perching. (C) Snapshots of the hanging perching experiment, capturing the process from takeoff to perching. (D) Snapshots of the grasping perching experiment, illustrating the sequence from takeoff to grasping, release, and perching. (E) Demonstration of the perching performance of the UAV on several special surfaces. The perching objects are, from left to right, 140 mm diameter cedar wood; 30 mm diameter round pole with a 10° angle to the horizontal; 70 mm diameter round pole with a 15° angle to the horizontal; and at the edge of a 30° slope.

To validate the performance of the BLCM in aerial scenarios, a series of perching and grasping experiments is conducted using UAV platforms (Figure 6A–C). The hardware and software configurations of the UAV are described in Methods, and flight position data are extracted from control log files. An aluminum 4040 frame is installed at the center of the test site, with cylindrical poles mounted as perching targets. In the standing perching experiment (Figure 6A; Figure S5 and Movie S3), the UAV takes off from the ground and targets a perch with a 70 mm diameter, positioned 3 m horizontally from the takeoff point and 1.8 m above ground. The maneuver is divided into two stages. First, the UAV ascends to ∼2.1 m before approaching the target. Second, when the center of the BLCM foot aligns with the perch axis, the landing sequence is initiated. Alignment is maintained until the perch is secured, at which point motor power is cut to allow passive holding (Figure 6B).

Hanging perching is evaluated in a subsequent experiment (Figure 6C; Movie S4). The UAV targets a 30 mm‐diameter pole located 2.5 m from the takeoff point. The procedure involves two steps: (i) the UAV first approaches to within ∼1 m of the target while maintaining clearance from surrounding structures, and (ii) it then ascends from below, aligning the foot center with the perch axis until contact is established. At this moment, the claw actively closes to secure suspension, and power is terminated. Notably, the BLCM allows autonomous selection of initial posture, enabling either hanging or standing perching depending on task requirements (Figure S6).

Dynamic aerial grasping is further assessed through a four‐step procedure (Figure 6D; Movie S5). The UAV first ascends to ∼1 m, hovers above a black foam target (40 × 40 × 100 mm^3^), then executes grasping and lifts the object to ∼1.5 m. The UAV subsequently transports and releases the object before returning to a safe landing. This sequence verifies the BLCM's ability to perform mid‐air manipulation with stable transitions between hovering, grasping, and release.

Finally, UAV perching is tested on specialized surfaces of varying geometry and texture (Figure 6E), demonstrating the adaptability of the BLCM across different environments. These experiments collectively confirm that the BLCM can be seamlessly integrated with UAVs, enabling both perching and grasping tasks in aerial environments. Such capabilities are critical for extending UAV endurance by allowing intermittent resting and for broadening operational utility in tasks such as inspection, delivery, and environmental monitoring.

## 3 Conclusion

This study introduces a novel BLCM, modeled on the hindlimb morphology of raptors, to address the longstanding challenge of enabling multirotor UAVs to perch and grasp effectively in outdoor environments [1]. BLCM combines three key components: a tensile paw structure, underactuated toes, and a collapsible leg. Together, these elements form a compact system weighing 420 g. With this configuration, UAVs are able to adopt two distinct perching strategies: standing and hanging, thereby accommodating targets with diverse shapes and surface geometries. In addition, BLCM provides active grasping capability, enabling the manipulation of external objects (Figure S7 and Movie S6). Notably, these functionalities are achieved with minimal reliance on sensors or complex control models, underscoring the robustness and efficiency of the mechanism in aerial applications. The BLCM embodies an optimized integration of established engineering concepts. It does not introduce new physical mechanisms or theoretical models, but instead emphasizes practical value in addressing real‐world UAV perching challenges.

Compared with existing UAV perching mechanisms, the BLCM demonstrates several unique advantages [8, 9, 10, 11, 12, 13, 29, 30, 31, 32, 33, 34, 35, 36, 37, 38]. First, it combines two perching modes within a single structure, whereas most reported designs are limited to either standing or hanging configurations (Table S1). Second, it integrates active grasping without the need for additional subsystems, offering multifunctionality beyond passive perching. Third, the design requires no high‐precision perception or control. Instead, it relies on strong passive adaptation to surface geometry, thereby reducing system complexity. While inspired by the zygodactyl foot structure of owls [54], BLCM advances beyond direct imitation through structural and functional innovations. For example, it enables passive perching on large‐diameter cylindrical branches as well as on flat ground. On 60–80 mm branches, it withstands tilts of at least ±5.5°. This capability supports UAVs weighing up to 1.2 kg. Increasing servo torque can further enhance the performance to support heavier platforms. Such improvement highlights the scalability of the design.

The rotatable toes of the BLCM further improve ground stability, allowing adaptation to irregular terrains (Figure S8 and Movie S7). Hanging perching is achieved by rotating the legs 180° and closing the toes via servo actuation. This allows UAVs to securely suspend from thin targets such as ropes and branches (Figure S9 and Movie S8). Minimization of energy costs during perching is achieved by experimentally optimizing torque thresholds. It is found that a stable suspension can be achieved at an output torque of only 1.2 kg·cm. This optimization reduces energy dissipation by ∼31.7 %, directly extending the endurance of UAV operations.

The grasping performance of the BLCM is evaluated using 30 objects of varied geometries, sizes, and surface textures. The system successfully grasps 28 objects (∼93 % success rate), particularly excelling with symmetrical or edge‐protruding geometries and soft surfaces. The two failed cases, involving objects without graspable protrusions, highlight geometry as a limiting factor but also suggest potential directions for further refinement of toe and claw design [61]. An important attribute of the BLCM is its scalability and ease of integration. The mechanism can be proportionally scaled to UAVs of different sizes, provided that the minimum spacing between wings exceeds the maximum leg width. Integration requires only a suitable mounting plate, without modification of the UAV airframe or flight control system. This universality, coupled with autonomous operation, facilitates broad applicability across UAV platforms.

By merging the perching strategies of both birds and bats within a single structure, BLCM overcomes key limitations of existing systems, which often demand restrictive perch geometries or are constrained to a single mode of operation. Beyond lab validation, it demonstrates significant potential for unstructured outdoor deployment, as its mechanical architecture enables stable perching even under environmental disturbances that impair high‐precision perception systems. As shown in this study, these advantages enhance UAV adaptability in complex environments, enabling versatile perching on diverse natural and artificial surfaces. For example, in disaster response or environmental monitoring, mode switching allows the utilization of various “perches of opportunity” (e.g., damaged structures, diverse tree species). Furthermore, the system's scalability accommodates varying payloads: the current version is optimized for a 1.2 kg takeoff weight, and increasing the servo torque allows for a higher grip force, potentially supporting heavier aerial manipulators for industrial inspection or cargo delivery tasks. Collectively, these capabilities open new opportunities for field applications, including environmental monitoring, infrastructure inspection, cargo delivery, and search‐and‐rescue missions where long‐duration deployment and interaction with the environment are required.

Although the proposed BLCM provides potential to enable robust perching and grasping for UAVs, there are some limitations and trade‐offs. First of all, the dual‐mode perching function is achieved with increased structural complexity and weight. The mechanism weighs approximately 430 g, about one quarter of the total UAV takeoff mass. This inevitably raises flight energy consumption and may slightly reduce endurance and payload capacity. Nevertheless, the benefits of dual‐mode perching meaningfully outweigh these drawbacks in unstructured environments. The mechanism enables stable standing and hanging perching without drone inversion, significantly improving adaptability and safety. Furthermore, long‐duration perching and power‐off idling can greatly reduce overall energy consumption compared with continuous hovering, thus compensating for the endurance loss caused by the extra weight. These results confirm the practical value of the proposed dual‐mode perching system for long‐duration UAV missions such as monitoring and inspection.

Future work may further refine the grasping capability for featureless objects, extend the design for heavier UAV platforms, and incorporate lightweight sensing modules for enhanced autonomy. Nonetheless, the present study establishes BLCM as a robust, efficient, and universal perching and grasping solution for UAVs, marking a step toward bioinspired aerial robotics capable of operating seamlessly in unstructured real‐world settings.

## 4 Methods

### 4.1 Manufacturing and Assembly

A total of seven BLCM prototypes were developed, with the present study reporting on the seventh iteration (Figure S10). Design parameters were iteratively refined through numerical optimization and repeated full‐scale prototype testing. Both manufacturing materials and assembly schemes underwent systematic evaluation prior to final selection to ensure structural integrity and functional reliability.

BLCM components were fabricated primarily by 3D printing using two resin materials—8200pro and T2000 (WENEXT)—via LCD laser fusion deposition. The connectors linking the UAV body and landing gear were manufactured from laser‐machined carbon fiber plates, ensuring a balance of strength and weight efficiency. Rubber‐based non‐slip pads (mesh EVA substrate, MILEQI) were affixed to the underside of the toes to improve surface friction during perching. Shafts and load‐bearing elements were made of bearing steel to withstand repeated mechanical stress. Tendon materials were determined through empirical testing of eight candidates, with Kevlar (0.5 mm, Dupont) selected for the passive tendons due to its high tensile strength and low elongation, and Dyneema (0.35 mm, Beadalon) adopted for the active tendons for its flexibility and durability.

Assembly of BLCM followed a bottom‐up sequence (Figure S11). First, the tensile foot structure was installed upon completion of the 3D‐printed parts. Eight tension springs (4 mm diameter, 0.4 mm wire diameter, 15 mm length) were then mounted to provide restoring force. Two bearings and torsion springs (0.7 mm wire diameter, 4 mm diameter, six turns, preset at 45°) were installed at each toe joint to enable compliant rotation. The toes were secured to the foot using an axle, after which linkages and servos were installed sequentially from the bottom upward. Finally, the tendons were threaded and fixed, with each pair of toes on the same side sharing a common tendon to ensure balanced force distribution and symmetric grasping. This modular manufacturing and assembly strategy not only facilitates rapid prototyping and iterative optimization but also ensures that BLCM can be scaled or adapted for integration with UAV platforms of different sizes.

### 4.2 Determination of the Length of Linkage 2

The length of linkage 2 is denoted as *L_14_*. The angle *θ_5_* can be obtained:

(5) $$\theta_{5}=\arccos\frac{{\left(R+L_{11}\right)}^{2}+R^{2}-{L_{10}}^{2}}{2R\left(R+L_{11}\right)}$$

Based on the geometric relationships of triangles OBC and OAD, it can be deduced that:

(6) $$\left\{\begin{array}{c} L_{13}/2=R\sin\left(\theta_{5}/2\right)\\ L_{12}/2=\left(R+L_{11}\right)\sin\left(\theta_{5}/2\right) \end{array}\right.$$

Furthermore, applying the cosine theorem to triangles ABC and ACD yields:

(7) $$\left\{\begin{array}{c} L_{11}^{2}=L_{10}^{2}+L_{13}^{2}-2L_{10}L_{13}\cos\theta_{6}\\ L_{11}^{2}=L_{10}^{2}+L_{12}^{2}-2L_{10}L_{12}\cos\theta_{6} \end{array}\right.$$

By combining Equations ((5), (6), (7)), the four unknowns *L_12_*, *L_13_*, *θ_6_* and *x_1_* can be solved. In addition, *L_14_* must satisfy the following condition:

(8) $$L_{14}^{2}=x_{1}^{2}+L_{12}^{2}-2x_{1}L_{12}\sin\left(\theta_{5}/2\right)$$

From the calculation, *L_14_* was determined to be no less than 37 mm, and a design value of 37 mm was therefore adopted.

### 4.3 Modeling the Relationship Between *M* and *γ*

The magnitude of the frictional force can be expressed as:

(9) $$\left\{\begin{array}{c} F_{fT}=\mu F_{N4}\\ F_{N4}d_{5}=F_{T}d_{6} \end{array}\right.$$

where *d_5_*, *d_6_* denote the characteristic parameters of the claw, *µ* is the coefficient of kinetic friction between the anti‐slip material and the branch, and *F_T_* is derived from the force analysis of the mechanism (Figure S1).

(10) $$\left\{\begin{array}{c} mg\cos\gamma -F_{a1}-F_{a3}=0\\ mg\sin\gamma +F_{a2}-F_{a4}=0\\ F_{a1}d_{4}/2+mgH_{2}\sin\gamma -F_{a3}d_{4}/2=0\\ F_{a1}d_{4}/2+F_{a4}H_{2}-F_{a3}d_{4}/2-F_{a2}H_{2}=0 \end{array}\right.$$

(11) $$\begin{array}{c} \left\{\begin{array}{c} F_{b1}-F_{b4}\cos\theta_{8}-F_{b3}\sin\theta_{8}=0\\ F_{b2}-F_{b4}\sin\theta_{8}+F_{b3}\cos\theta_{8}-F_{T}=0\\ \left(F_{b4}\cos\theta_{8}+F_{b3}\sin\theta_{8}\right)L_{9}\sin\theta_{4}-\left(F_{b4}\sin\theta_{8}+F_{b3}\cos\theta_{8}\right)\\ \;\times \;L_{9}\cos\theta_{4}-F_{T}L_{9}\cos\theta_{4}=0\\ F_{c1}-F_{c4}\cos\theta_{8}-F_{c3}\sin\theta_{8}=0\\ F_{c2}-F_{c4}\sin\theta_{8}+F_{c3}\cos\theta_{8}-F_{T}=0\\ \left(F_{c4}\cos\theta_{8}+F_{c3}\sin\theta_{8}\right)L_{9}\sin\theta_{4}-\left(F_{c4}\sin\theta_{8}+F_{c3}\cos\theta_{8}\right)\\ \;\times \;L_{9}\cos\theta_{4}-F_{T}L_{9}\cos\theta_{4}=0 \end{array}\right. \end{array}$$

where *H_2_* is the distance between the load and the center of the joint linkage above the quadrilateral linkage, and *m* is the load mass. Furthermore, when the system is in equilibrium, force and moment balance exist in both the X and Y directions.

(12) $$\begin{array}{c} \left\{\begin{array}{c} Mg\cos\gamma +2F_{N4}\cos\theta_{7}-F_{N2}\cos\theta_{5}-F_{N3}\cos\theta_{5}-F_{N1}\cos\theta_{5}\\ \;+\left(F_{f1}\sin\theta_{5}-F_{f3}\sin\theta_{5}\right)=0\\ Mg\sin\gamma -F_{N1}\sin\theta_{5}+F_{N3}\sin\theta_{5}\\ \;-\left(F_{f2}-F_{f1}\cos\theta_{5}-F_{f3}\cos\theta_{5}+2F_{fT}\cos\theta_{7}\right)=0\\ MgH_{1}\sin\gamma +F_{N1}R\cos\theta_{5}-F_{N3}R\cos\theta_{5}-2F_{f1}R\sin^{2}\theta_{5}\\ \;-\;2F_{f3}R\sin^{2}\theta_{5}-2F_{fT}R\left(1+\cos\theta_{7}\right)=0 \end{array}\right. \end{array}$$

The relationship between *M* and *γ* at the instant of equilibrium failure under a given load can be obtained by combining Equations (8) and (12).

### 4.4 Power Optimization Solutions

A digital power test module (INA219, DFRobot Gravity) was employed to evaluate the power consumption of the BLCM during grasping. An Arduino microcontroller (UNO R3, Arduino) was used for data acquisition, and the collected signals were transmitted to a computer for processing. Data visualization and subsequent analysis were performed using Python, enabling accurate characterization of power and energy consumption during grasping tasks.

### 4.5 Control Scheme of BLCM

BLCM is actuated by six servomotors: four single‐axis servos (HL‐3606‐C0002, Feetech) driving the tendons and two dual‐axis servos (HL‐3606‐C0001, Feetech) serving as joint actuators. Servo control was implemented via an ESP‐32 development board (ESP32‐S3‐DEV‐KIT, Waveshare).

The BLCM control logic is organized into three operational modes. In Standing Perching Mode, servos are not actuated. The system only ensures joint motors are powered and electronically locked for stability. For Hanging Perching Mode, a strict timing sequence is executed: joint servos rotate the legs by 180°, followed by the synchronous actuation of four tendon servos to achieve suspension. The use of a shared tendon for each pair of toes inherently balances the grasping force. In Grasping Mode, a force‐position hybrid control strategy is implemented, wherein a predefined position command ensures target enclosure, followed by a torque‐limiting phase to prevent mechanical damage. Additionally, a power‐on reset routine is executed before each mission to eliminate tendon slack, ensuring consistent and repeatable performance.

The rotation of each servo (360°) was discretized into 4096 steps, enabling fine‐grained positional control by setting step increments. To ensure consistent tendon tension, manual pre‐adjustments were made prior to each experimental trial. Additionally, the tendon‐actuating servos were programmed to automatically return to their initial positions at power‐up, ensuring repeatable starting conditions. Joint servo positions were fixed by the control program and remained unaltered throughout operation.

Servo angular velocity was programmatically defined as 150 steps per second, equivalent to ∼13.17° per second. The control board operated at 7 V, powered by a 2200 mAh lithium‐ion battery (2S, HUAYPG), providing stable power for all actuation tasks (Figure S12).

### 4.6 Calibration of Strain Gauges

Thin‐film pressure sensors (FSR400, Interlink Electronics) were employed as strain gauges. For calibration, each sensor was mounted on a pressure instrument (HP1000N, HANDPI), while the resistance knob of the signal converter module was adjusted under applied loads. Calibration was considered successful when the sensor readings were within ±5 % of the reference values provided by the pressure instrument.

To ensure accurate force transmission, small discs with an area equivalent to that of the sensing surface were mounted on the transducer. Identical discs were also mounted symmetrically on the opposite side to balance the applied load. All sensors were connected to a four‐channel signal converter module (WAAAX, China), which transmitted aggregated data to a PC for visualization and analysis. The experimental setup is illustrated in Figure S13.

### 4.7 Design of Grasping Experiments

Objects were placed on a flat table, and grasping trials were conducted using BLCM with the servo output torque fixed at 1.2 kg·cm. For each object, three independent trials were performed. A trial was considered successful if the object could be securely held for more than 10 s, and overall success was defined as at least two successful attempts out of three [61].

Grasping was performed exclusively from the top in order to replicate the UAV operation. This setup simulated the natural approach trajectory of the vehicle during aerial manipulation. This protocol ensured consistency across trials and provided a standardized basis for evaluating the grasping performance of BLCM.

### 4.8 UAV Hardware Configuration

The UAV platform used in this study had a wheelbase of 450 mm and was equipped with a Pixhawk 6C flight control unit (Holybro) and a Jetson Orin Nano onboard computer (8G; NVIDIA). The propulsion system consisted of 2312 motors (DONGXINGWEI) controlled by Goodwin 20A electronic speed controllers (ESCs; HOBBYWING), paired with 9‐inch propellers (9450, YH). Power was supplied by a 5300 mAh lithium polymer battery (4S, ACE). The overall hardware configuration is shown in Figures S14 and S15.

### 4.9 Lab‐Based Experiments on Perching and Grasping UAVs

Indoor experiments were conducted using a motion capture system (ChingMu, China) as the core positioning reference module. Fixed markers mounted on the UAV body were tracked in real time by the cameras, which output raw positioning data streams containing 3D coordinates, Euler angles, and instantaneous velocities at a sampling frequency of 120 Hz. The spatial data were transmitted to the onboard computer via Ethernet for further processing.

A real‐time processing framework was implemented to enhance data quality and usability. First, noise suppression was performed using a Kalman filter. The motion capture data in the world coordinate frame was then transformed into relative position information in the UAV body coordinate system. Timestamp synchronization and data formatting were achieved through a ROS (Robot Operating System) node. To improve accuracy and compensate for system delays, high‐frequency inertial measurement unit (IMU) data were fused with the motion capture data using an Extended Kalman Filter (EKF). The EKF, a recursive least‐squares filter, effectively mitigated motion‐capture transmission delays and IMU drift. The fused state estimates were provided to the UAV's flight control system as inputs to a PID controller. This controller adjusted the PWM outputs of the brushless motors and servo rudders in real time, ensuring accurate trajectory tracking and dynamic correction of flight deviations. As a result, the UAV demonstrated the ability to maintain stable hovering with a position error ≤ 1 cm in complex indoor environments without GPS signals.

The UAV was operated via manual remote control, with a mobile ground station used to monitor its flight status. Position data of all objects were logged through the UAV flight control system for subsequent analysis.

## Funding

This work was supported in part by the Shanghai Municipal Science and Technology Major Project under grant 2021SHZDZX0103, and the Key Project of Comprehensive Prosperity Plan of Fudan University under grant XM06231744.

## Conflicts of Interest

The authors declare no conflicts of interest.

## Supporting information




**Supporting File 1**: advs74831‐sup‐0001‐SuppMat.docx.

**Supporting File 2**: advs74831‐sup‐0002‐MovieS1.mp4

**Supporting File 3**: advs74831‐sup‐0003‐MovieS2.mp4.

**Supporting File 4**: advs74831‐sup‐0004‐MovieS3.mp4.

**Supporting File 5**: advs74831‐sup‐0005‐MovieS4.mp4

**Supporting File 6**: advs74831‐sup‐0006‐MovieS5.mp4.

**Supporting File 7**: advs74831‐sup‐0007‐MovieS6.mp4

**Supporting File 8**: advs74831‐sup‐0008‐MovieS7.mp4.

**Supporting File 9**: advs74831‐sup‐0009‐MovieS8.mp4.

## Data Availability

The data that support the findings of this study are available in the supplementary material of this article.
