# Supplementary material for: Bioinspired Adaptive Leg‐Claw Enables Robust Perching and Grasping for UAVs
Source: Adv Sci (Weinh). 2026 Mar 13;13(30):e23518. doi: 10.1002/advs.202523518 (PMC13248824; doi:10.1002/advs.202523518)
Supplement: Supplementary file 1 — Supporting File 1: advs74831‐sup‐0001‐SuppMat.docx. [file ADVS-13-e23518-s004.docx]

Supplementary Materials for

**Bioinspired Adaptive Leg-Claw Enables Robust Perching and Grasping for UAVs**

Tianyu Cheng, Shaokun Wang, Jiehan Zou, Guiwen Shi, Zhongxue Gan*,

I-Ming Chen, Guo-Niu Zhu*

*Corresponding author. Email: guoniu_zhu@fudan.edu.cn

**This file includes:**

Supplementary Text

Figures S1 to S15

Tables S1 to S4

Movies S1 to S8

Supplementary Text


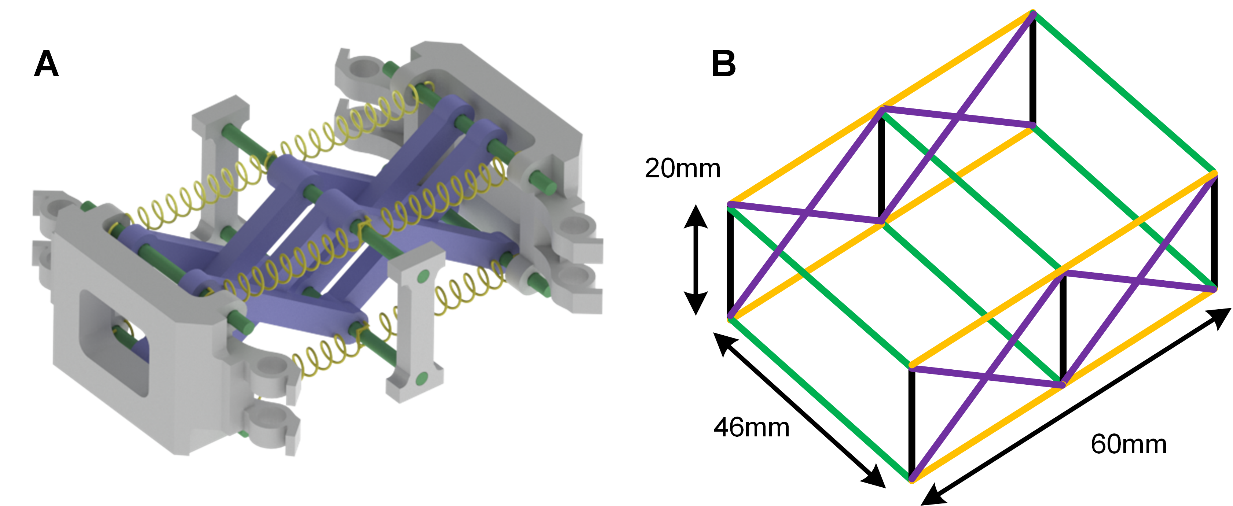


Figure S1. Visualization of the tension structure. (A) Rendered model of the tension structure. (B) Simplified schematic diagram.


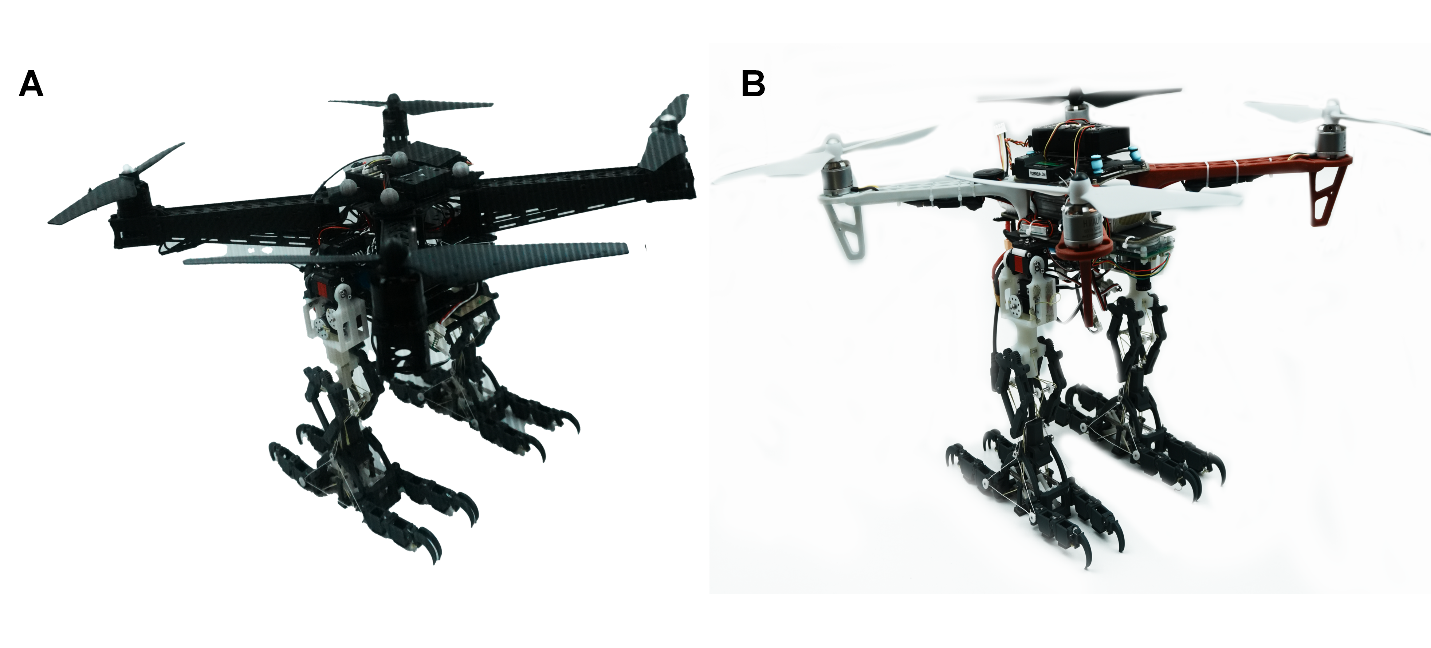


Figure S2. UAV platforms used in this study. (A) F450 quadrotor employed for motion-capture experiments. (B) F450 quadrotor employed for static testing.


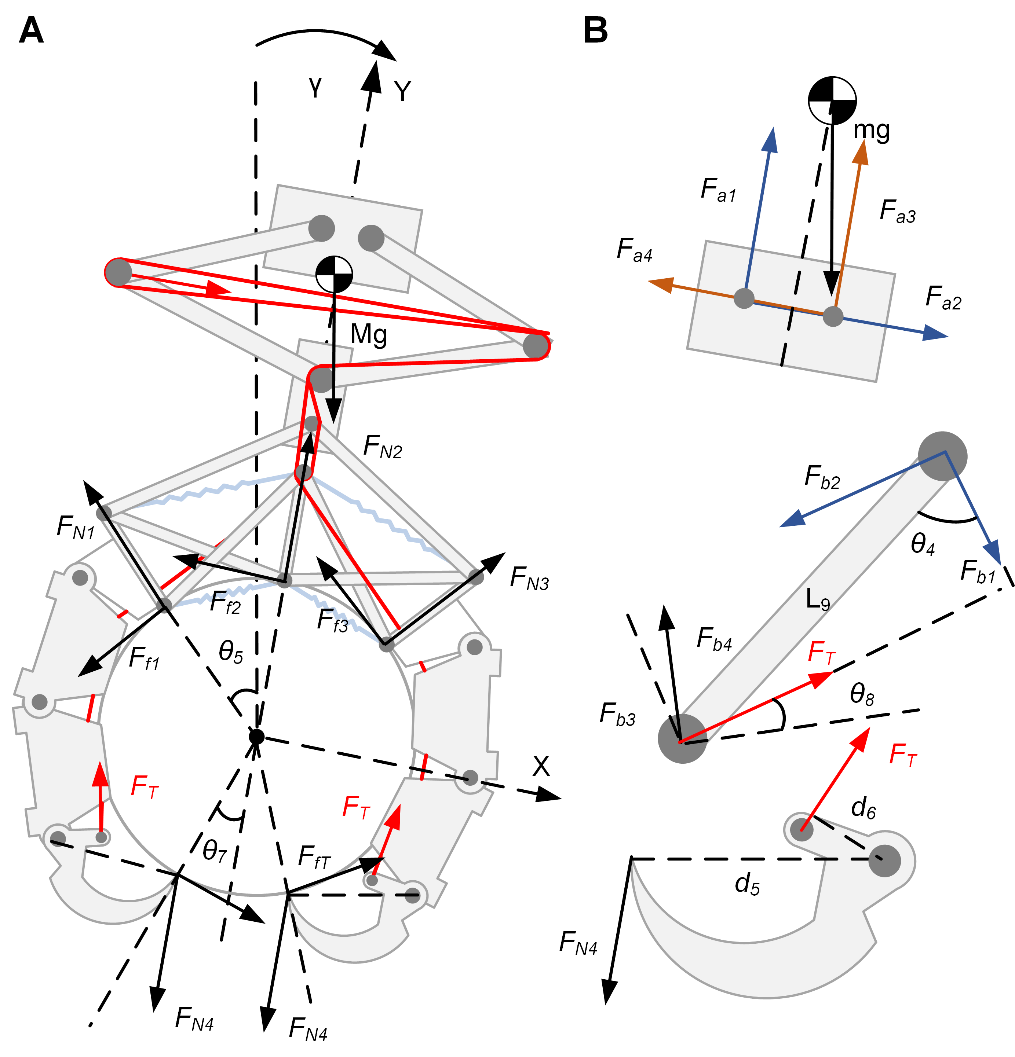


Figure S3. Force analysis of the BLCM during standing perching. (A) Overall force distribution. (B) Forces acting on key components.


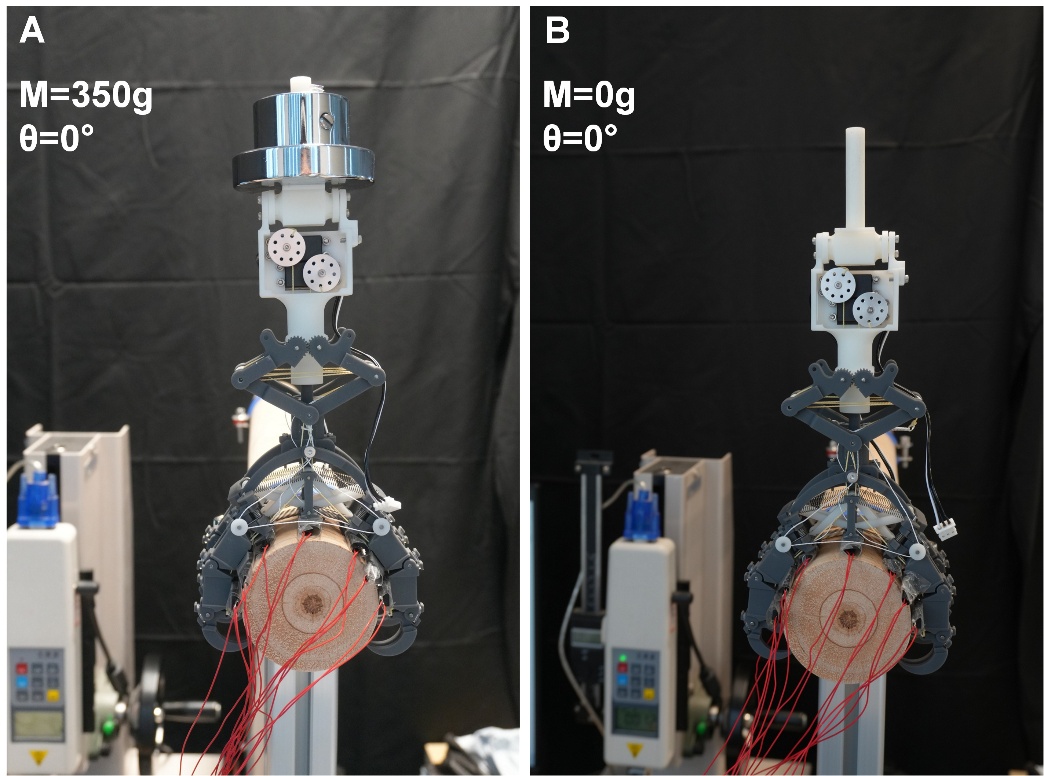


Figure S4. Stress test experiments under different loading conditions. (A) Applied load: 350 g. (B) No load (0 g).


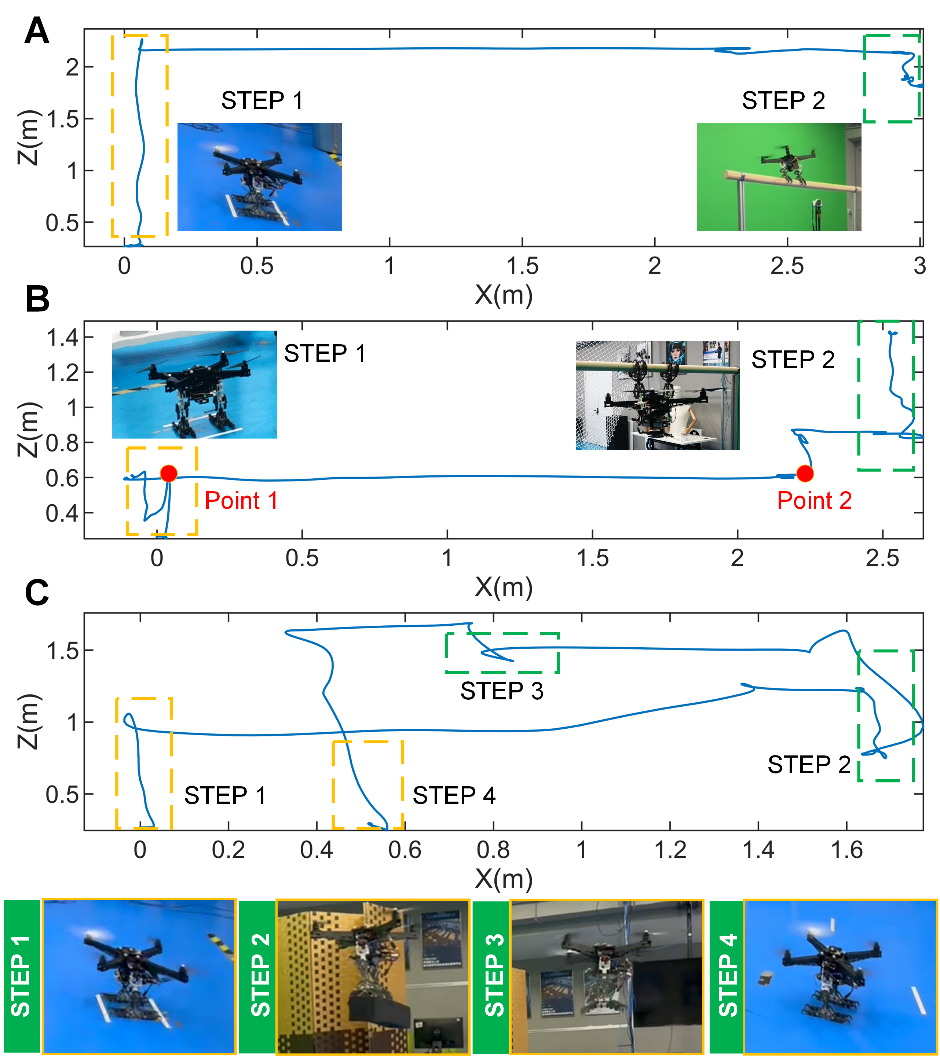


Figure S5. Two-dimensional trajectories of UAV flight experiments. (A) Standing perching. (B) Hanging perching. (C) Grasping.


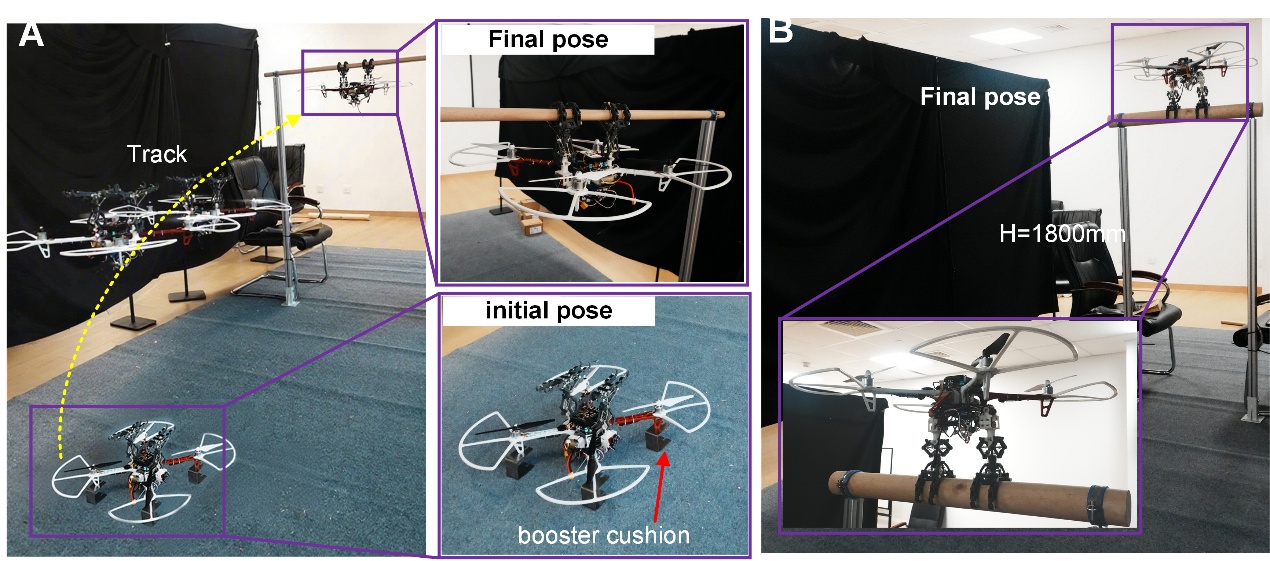


Figure S6. Supplementary indoor flight experiments. (A) Hanging takeoff leading to hanging. (B) Hanging takeoff leading to standing.


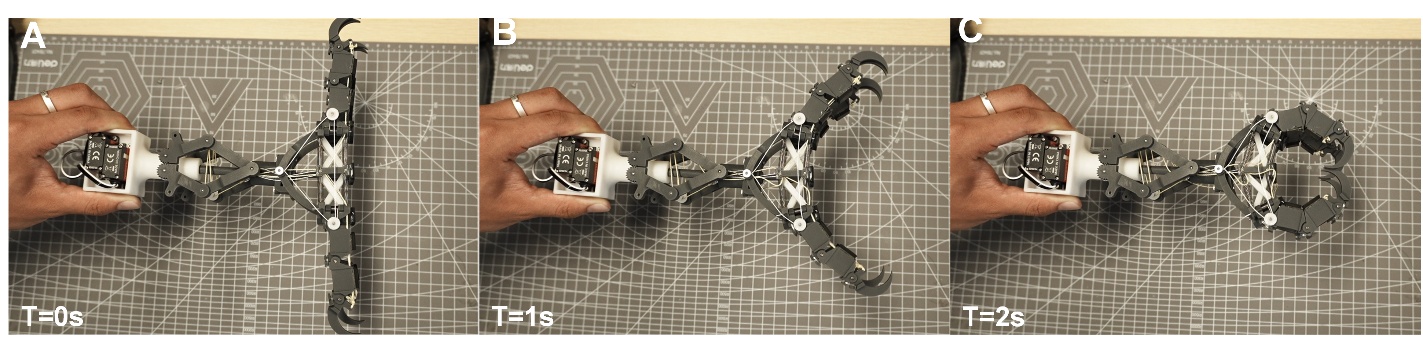


Figure S7. Demonstration of the BLCM active gripping functionalities. (A) T=0s, BLCM in the initial state. (B) T=1s, BLCM closing. (C) T=2s, BLCM fully closed.


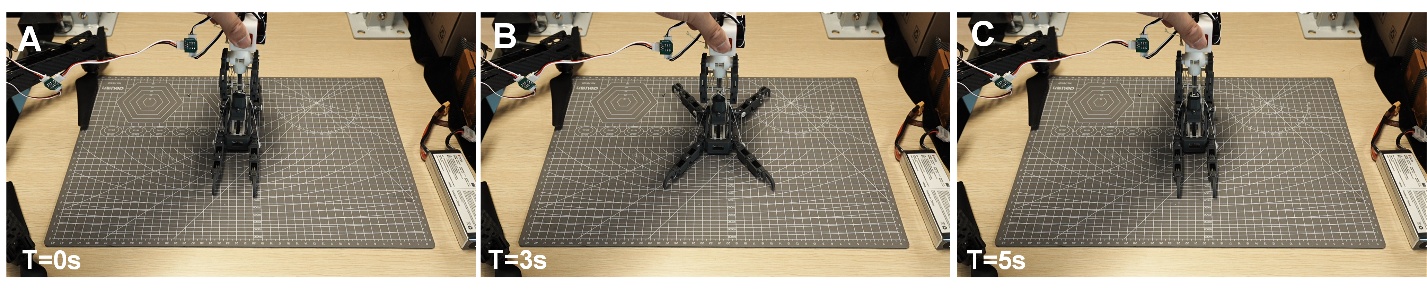


Figure S8. Demonstration of the active opening and closing functionalities of BLCM toes. (A) T=0s, BLCM in the initial state. (B) T=3s, BLCM toes fully open. (C) T=5s, BLCM toes fully closed.


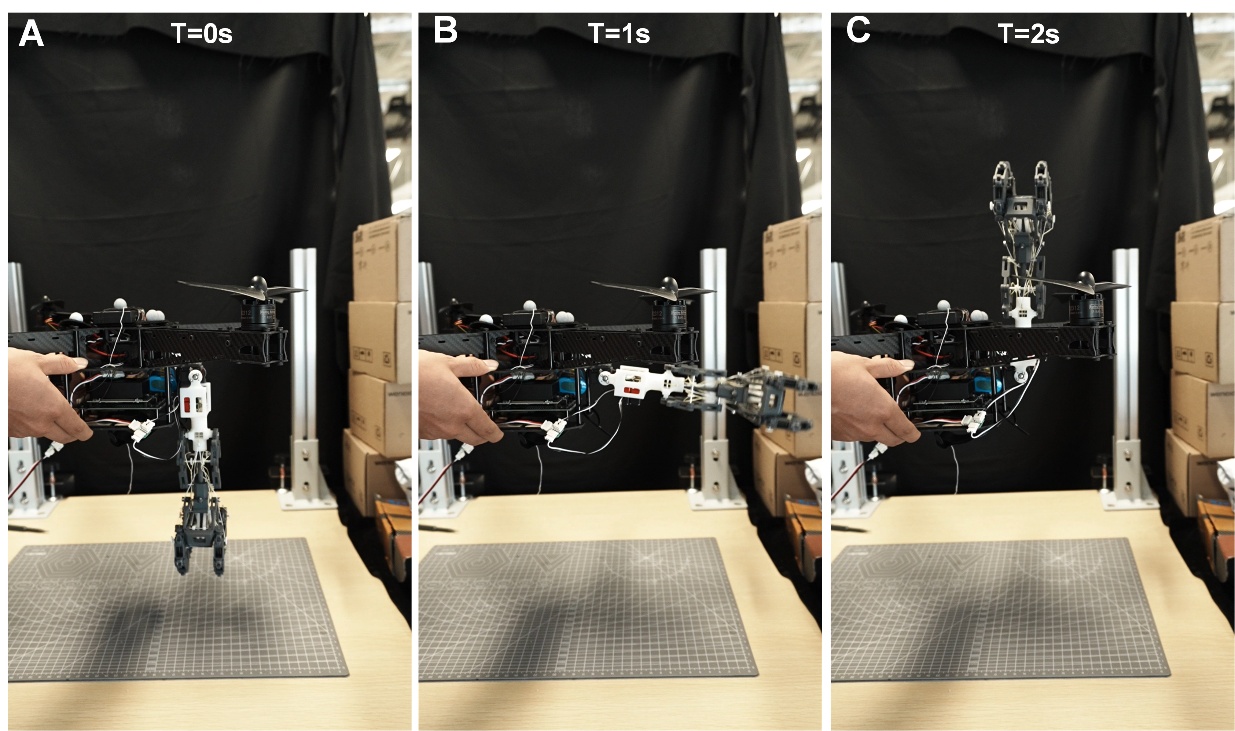


Figure S9. Demonstration of the BLCM thigh rotation functionalities. (A) T=0s, initial state. (B) T=1s, thigh rotated ~90°. (C) T=2s, thigh rotated to inverted posture.


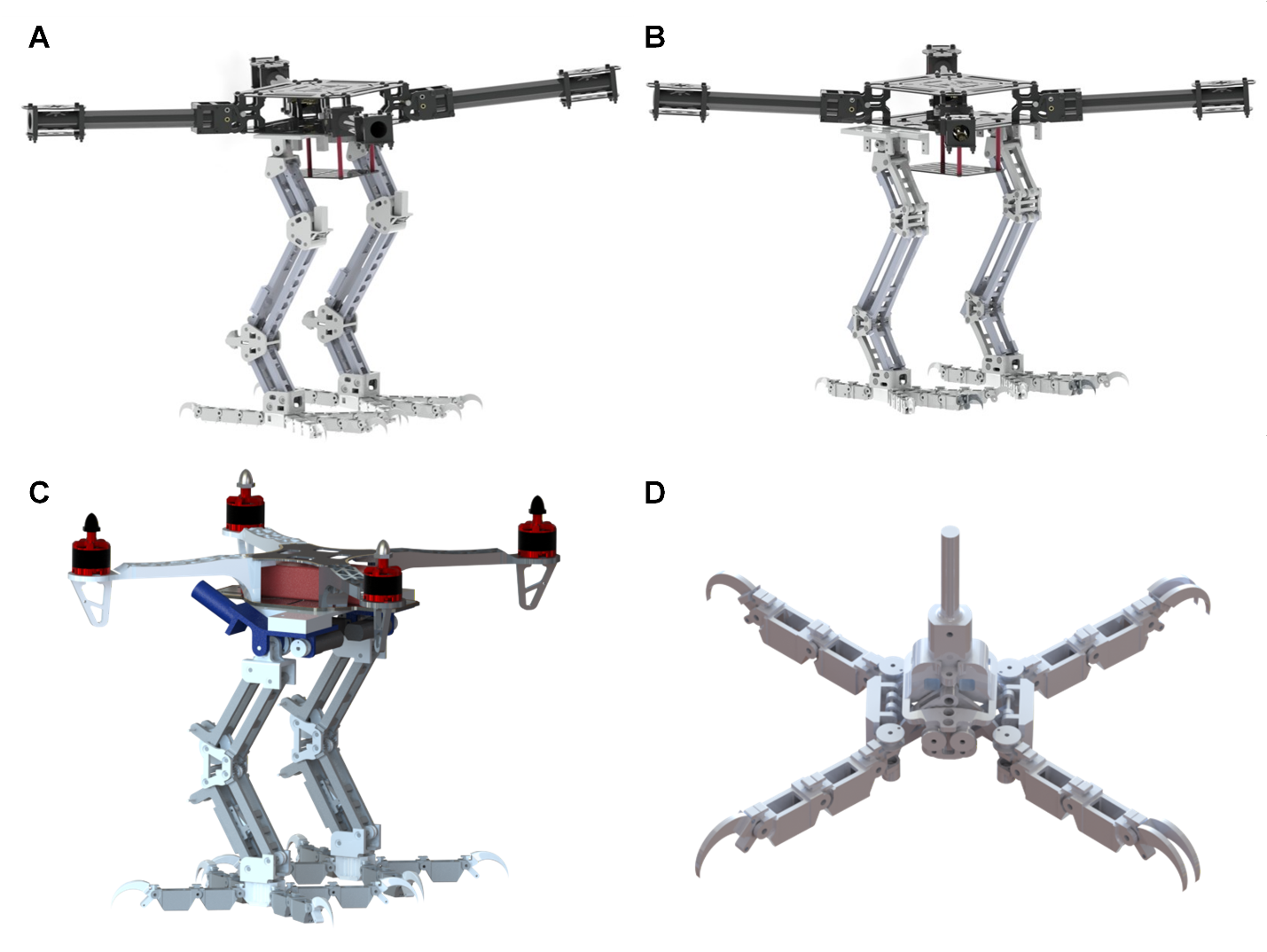


Figure S10. Several early version structural renderings. (A) Second-generation version (November 2024). (B) Third-generation version (December 2024). (C) Fifth-generation version (February 2024). (D) Sixth-generation version (April 2025).


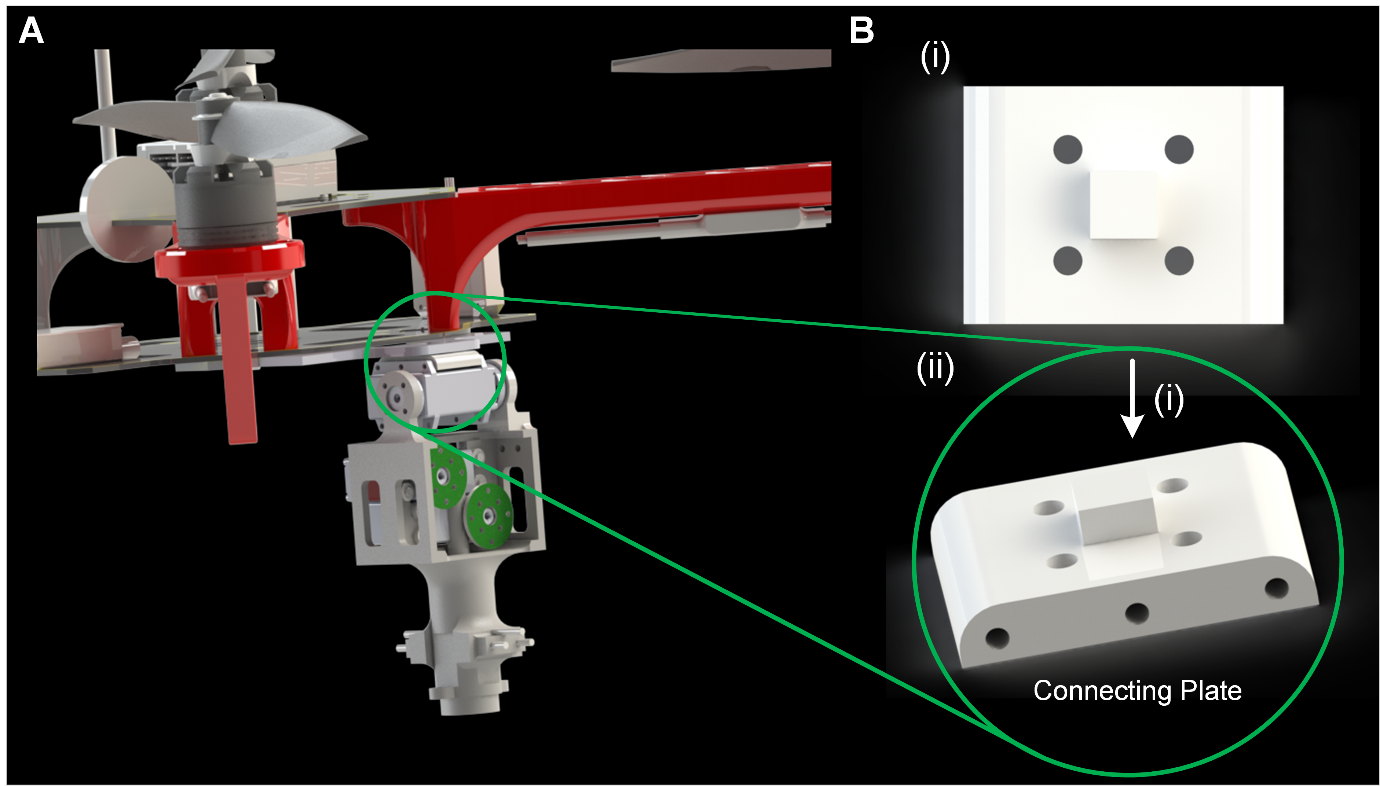


Figure S11. Connection between the BLCM and UAV platform. (A) Structural schematic of the connection point. (B) Design of the connection component.


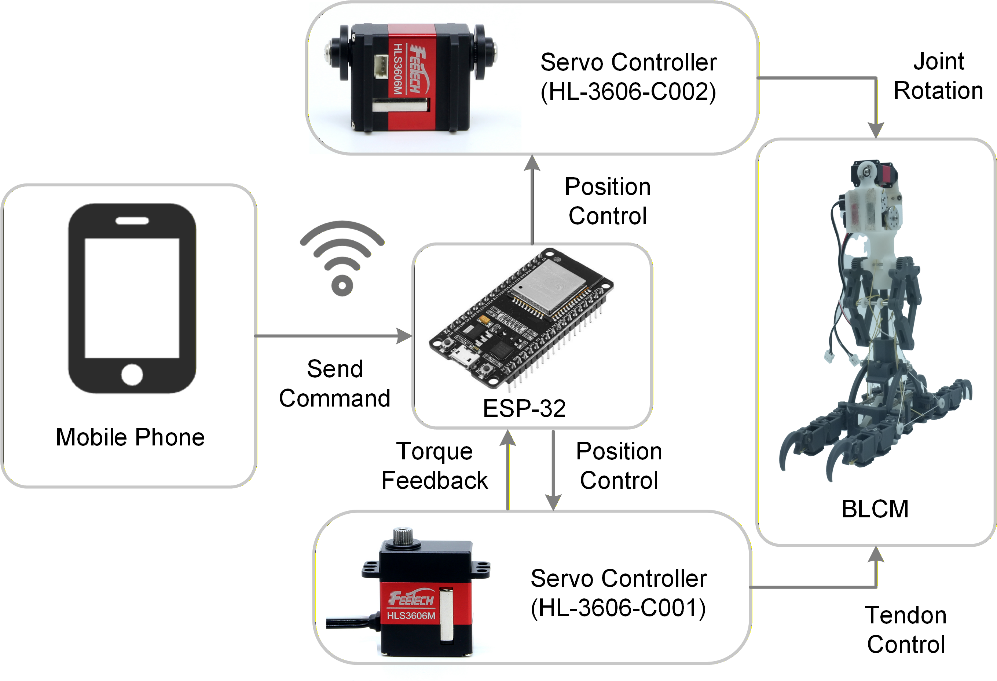


Figure S12. Control scheme of the BLCM.


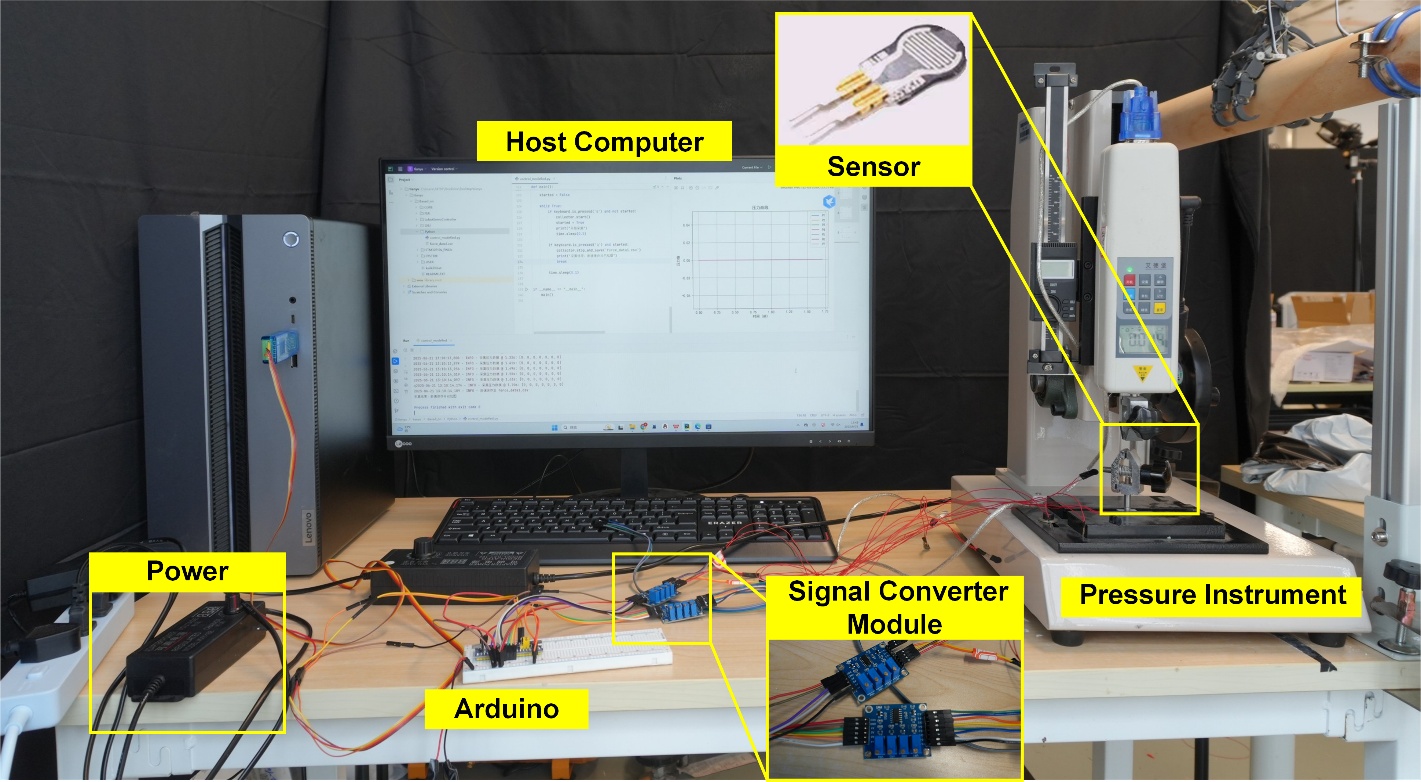


Figure S13. Calibration of thin-film pressure sensors and Python-based data acquisition.


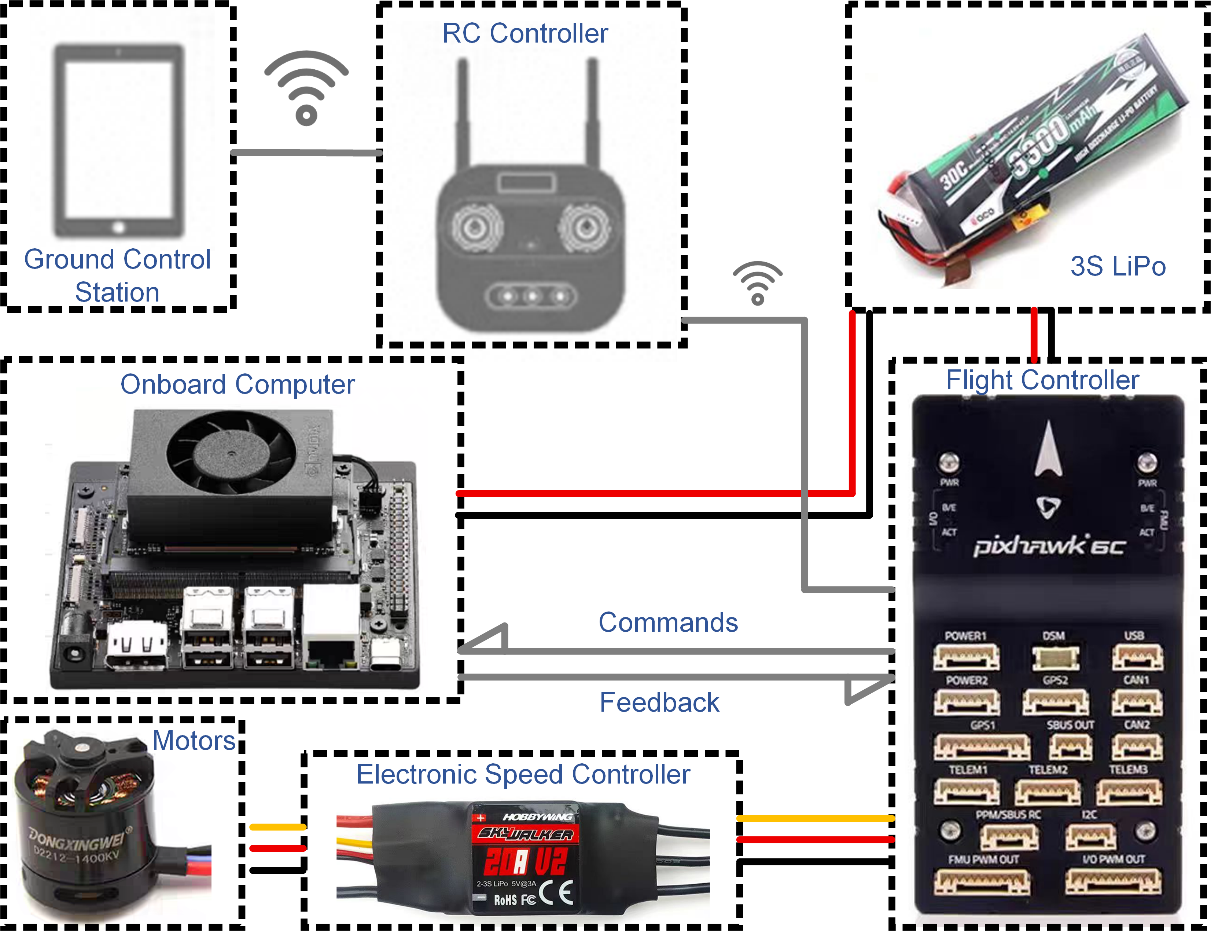


Figure S14. Hardware setup for UAV motion capture experiments


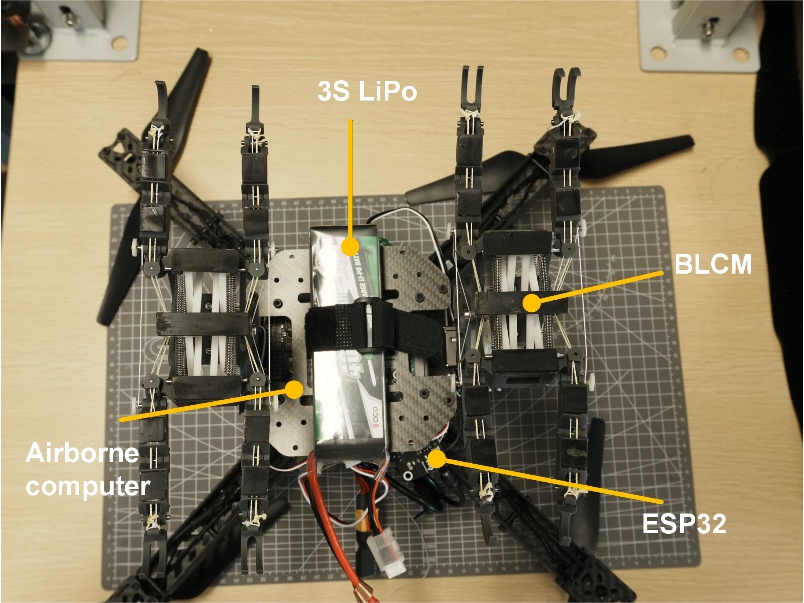


Figure S15. Underside view of the UAV hardware layout. A 3S LiPo battery (3300 mAh) is mounted at the bottom, with the ESP32 control board and onboard computer positioned directly behind it.

Table S1. Comparison of parameters of grasping-based perching mechanisms for UAVs

|  | **Mass (g)** | **Mass** **fraction (%)** | **Active Grasping** | **Standing** | **Hanging** | **Years** |
| --- | --- | --- | --- | --- | --- | --- |
| Perching Landing Gear System [11] | 178.4 | 10 | × | √ | × | 2019 |
| Compliant Bistable Gripper [37] | 9 | 25 | × | × | √ | 2019 |
| Passively Adaptive Grapple [33] | 32 | 1.8 | × | × | √ | 2019 |
| Passive Adaptive Robot Hand [23] | 551 | / | √ | √ | × | 2019 |
| SNAG [19] | 250 | 33.3 | × | √ | × | 2021 |
| Passive Dynamic Bioinspired Gripper [34] | 300 | 14.3 | × | √ | √ | 2023 |
| Avian-Inspired Claws [29] | 114 | 14 | × | × | × | 2024 |
| Bat-like Perching UAV [35] | / | / | × | × | √ | 2024 |
| Bionic Bird Claw [3] | 177.72 | / | √ | √ | × | 2024 |
| Passive Mechanism [31] | 400 | 20.9 | × | √ | × | 2024 |
| RAVEN [58] | 216 | 36 | × | × | × | 2024 |
| Bistable Soft Gripper [30] | 536 | 26.8 | √ | √ | × | 2025 |
| Bistable Robotic Gripper [38] | 132.21 | 24.12 | × | × | √ | 2025 |
| GPALM [9] | / | / | √ | √ | × | 2025 |
| **BLCM** | **420** | **24.7** | **√** | **√** | **√** | **/** |

Note: “√” means having this ability, while “×” means not giving this ability. ‘Mass’ refers to the total mass of the mechanism mounted on the UAV, while ‘Mass fraction’ denotes the proportion of the landing gear system relative to the total mass of the UAV. Our mechanism achieves the most comprehensive integration of perching and grasping functionalities, while preserving a balanced and efficient overall mass distribution.

Table S2. Dimensions of key components

| **Name** | **L×H×W**  **(mm×mm×mm)** |
| --- | --- |
| Compression Linkage | 40×8×5 |
| Pedestal | 16×16×63 |
| Bent Linkage | 37×6.5×5 |
| Claw | 25×8×18 |
| Phalanx 1 | 24×12×18 |
| Phalanx 2 | 24×12×18 |
| Linkage | 31×4×3 |
| Thigh | 92.5×39×44 |
| Steering Wheel | 18×5×18 |

Table S3. Experimental data

| **Diameter (mm)** | **Load (g)** | | | | | | | |
| --- | --- | --- | --- | --- | --- | --- | --- | --- |
|  | **0** | **50** | **100** | **150** | **200** | **250** | **300** | **350** |
| 60 | 19.15 | 15.30 | 11.20 | 9.50 | 9.10 | 8.65 | 8.45 | 8.45 |
| 70 | 21.75 | 14.50 | 11.25 | 9.65 | 9.1 | 8.55 | 8.35 | 8.35 |
| 80 | 21.95 | 15.25 | 11.35 | 11.25 | 10.30 | 9.60 | 8.00 | 6.95 |
| 90 | 22.5 | 15.7 | 13.85 | 13.05 | 10.10 | 6.20 | 5.80 | 5.30 |
|  | **400** | **450** | **500** | **550** | **600** | **650** | **700** | **750** |
| 60 | 8.00 | 7.80 | 7.65 | 7.2 | 7 | 6.85 | 6.75 | 6.75 |
| 70 | 7.80 | 7.52 | 7.60 | 6.95 | 6.05 | 5.55 | 5.50 | 5.20 |
| 80 | 6.80 | 6.55 | 6.10 | 5.70 | 5.55 | 5.30 | 5.21 | 4.75 |
| 90 | 4.60 | 4.25 | 3.90 | 3.75 | 3.70 | 3.70 | 3.50 | 3.05 |

Table S4. Object features and experimental results of grasping experiments

| **Name of Object** |  | **Weight (g)** | **L×H×W (mm×mm×mm)** | **Hard or Soft?** | **Successful capture? (Yes/No)** |
| --- | --- | --- | --- | --- | --- |
| Cube1 |  | 43 | 60×60×60 | Soft | Yes |
| Cube2 |  | 118 | 70×70×70 | Hard | Yes |
| Rubber ball |  | 102 | 120×120×120 | Soft | Yes |
| Triangular prism |  | 18 | 50×50×50 | Hard | No |
| Cylinder |  | 22 | 50×50×50 | Soft | Yes |
| Canned beverage |  | 302 | 55×55×80 | Hard | Yes |
| Sphere |  | 16 | 45×45×45 | Hard | Yes |
| Mango |  | 150 | 122×57×62 | Soft | Yes |
| Plate |  | 85 | 190×190×28 | Hard | Yes |
| Hexagonal prism1 |  | 49 | 111×75×75 | Soft | Yes |
| Hexagonal prism2 |  | 42 | 133×40×45 | Soft | Yes |
| Thin plate |  | 36 | 160×68×7 | Hard | No |
| Tape roll |  | 210 | 108×108×48 | Soft | Yes |
| Pliers |  | 221 | 150×54×17 | Hard | Yes |
| Basket |  | 351 | 253×347×155 | Hard | Yes |
| Foam mat |  | 143 | 400×400×70 | Soft | Yes |
| Water bottle1 |  | 80 | 40×40×150 | Hard | Yes |
| Water bottle2 |  | 350 | 40×40×150 | Hard | Yes |
| Camera gimbal |  | 355 | 127×170×127 | Hard | Yes |
| Hooked weight |  | 1000 | 45×45×55 | Hard | Yes |
| Cup |  | 60 | 78×78×105 | Hard | Yes |
| Stuffed toy |  | 420 | 380×180×196 | Soft | Yes |
| Shoes |  | 230 | 250×105×112 | Soft | Yes |
| Book |  | 408 | 200×150×20 | Soft | Yes |
| Vise |  | 2000 | 140×180×80 | Hard | Yes |
| Clothes |  | 100 | 150×150×60 | Soft | Yes |
| Apple |  | 321 | 100×100×100 | Hard | Yes |
| Banana |  | 264 | 180×42×45 | Soft | Yes |
| Toilet paper roll |  | 125 | 120×120×160 | Soft | Yes |
| Keychain |  | 180 | 45×55×12 | Hard | Yes |

Movie S1. BLCM active grasping experiment: some successful cases.

Movie S2. BLCM active grasping experiment: some failure cases.

Movie S3. Indoor UAV standing perching experiment.

Movie S4. Indoor UAV hanging perching experiment.

Movie S5. Indoor UAV grasping experiment.

Movie S6. BLCM active opening and closing.

Movie S7. Active adjustment of the BLCM toe angle.

Movie S8. Changing perching posture by flipping the leg.
